# Mitigating delay due to capacity drop near freeway bottlenecks: Zones of influence of connected vehicles

**DOI:** 10.1371/journal.pone.0301188

**Published:** 2024-06-05

**Authors:** Kshitij Jerath, Vikash V. Gayah, Sean N. Brennan

**Affiliations:** 1 Department of Mechanical Engineering, University of Massachusetts, Lowell, MA, United States of America; 2 Department of Civil and Environmental Engineering, The Pennsylvania State University, State College, PA, United States of America; 3 Department of Mechanical Engineering, The Pennsylvania State University, State College, PA, United States of America; Southwest Jiaotong University, CHINA

## Abstract

We present a novel perspective on how connected vehicles can reduce total vehicular delay arising due to the capacity drop phenomenon observed at fixed freeway bottlenecks. We analytically determine spatial regions upstream of the bottleneck, called *zones of influence*, where a pair of connected vehicles can use an event-triggered control policy to positively influence a measurable traffic macrostate, e.g., the total vehicular delay at bottlenecks. These analytical expressions are also able to determine the boundaries (called *null* and *event horizons*) of these spatial extents, outside of which a connected vehicle cannot positively influence the traffic macrostate. These concepts can help ensure that information is disseminated to connected vehicles in only those spatial regions where it can be used to positively impact traffic macrostates. Some scenarios examined in this study indicate that communication between connected vehicles may be required over a span of several kilometers to positively impact traffic flow and mitigate delays arising due to the capacity drop phenomenon.

## 1 Introduction

The phenomenon of capacity drop manifests as a reduction in capacity at or near a bottleneck, when the upstream flow exceeds the rated capacity of the bottleneck. In this scenario, the flow through the bottleneck may drop below its rated capacity, and various empirical studies have estimated this capacity drop to be between 3% to 18% [1]. The consequence of the capacity drop phenomenon is increased congestion and delays at bottlenecks. This often has direct measurable impacts, such as in terms of decreased productivity due to person-hours lost, increased costs due to fuel wastage, and adverse environmental impacts due to increased emissions [2]. As a result, significant research effort has been directed towards better understanding the issue of capacity drop. For example, the existence of a two-capacity phenomenon at the bottlenecks has been discussed in [3], and a methodology to estimate capacity drop at merging highway sections has been developed in [4]. The notion that such a drop may occur over spatially extended regions rather than at the bottleneck point has also been studied [5, 6]. While there is ongoing debate regarding the root cause of the phenomenon, several approaches have been proposed as potential mechanisms for mitigating capacity drop and the resulting congestion. For example, recent works have discussed the use of variable speed limits (VSL) and connected vehicles to control capacity drop [7–10].

In this paper, we build upon these works by using the well-known Lighthill-Whitham-Richards (LWR) model to demonstrate that knowledge of the *spatial location* of connected vehicles is an important factor for successfully mitigating capacity drop at fixed freeway bottlenecks. Specifically, given the increasing ability to inform drivers about nearby traffic conditions using vehicle-to-everything (V2X) technologies, it becomes natural to ask the following questions: Can an individual driver use such information to positively affect traffic flow? More importantly, which drivers in a traffic network have the most influence on traffic flow, i.e. *where and to whom should this information be delivered*? To answer these questions, the authors introduce two new concepts with regards to connected vehicles—the zone of influence (ZOI), and the null and event horizons—and demonstrate their utility in the context of mitigating capacity drop. The *zone of influence* of a connected vehicle refers to the region of a roadway within which the vehicle can exert control and, as a consequence, positively influence the macroscopic traffic flow. The *null* and *event horizons* act as the boundary demarcating the ZOI on the roadway from other locations where actions by connected vehicles have no positive macroscopic impact on traffic flow.

The **primary objective** of this paper is to provide an analytical introduction to the novel concept of zones of influence of connected vehicles within a specific traffic flow context: the phenomenon of capacity drop observed near fixed freeway bottlenecks. A **secondary objective** is provide this introduction in a manner that enables researchers to expand upon these concepts and apply them across a diverse set of problems and modeling approaches [11]. For example, the concept of a ZOI may be applicable to tasks such as positioning of variable-message signs, designing appropriate coverage areas for V2X elements, location of on- or off-ramps, identifying platoon merging or splitting zones that do not disrupt traffic flow, and even enabling continuous traffic control via connected vehicles. In each of these problems, knowledge of the ZOI may prove to be vital to improve system performance.

The main contributions of this work are as follows:

(a) We have introduced the novel concept of **zones of influence (ZOI) of connected vehicles (CVs)**. We have shown the existence of the ZOI in a traffic bottleneck scenario that is accompanied by a capacity drop.
(b) Equally as importantly, we have identified **analytical expressions that determine the spatial limits of influence** of CVs on specific traffic flow characteristics. This concept of event horizons and null horizons to quantify where CVs begin and cease to have positive influence is novel in the field.
(c) To demonstrate this concept of ZOI, we have also developed the notion of a **user-defined traffic macrostate**, which is the high-level traffic descriptor that the connected vehicles are attempting to influence. In this work, the traffic macrostate is given by the total vehicular delay experienced by all vehicles that have been delayed at the bottleneck.
(d) We have also developed a novel approach to use the **concept of event-triggered control policies for CVs** from the perspective of influencing traffic flow macrostate. To our knowledge, the notion of bottleneck detection-based event triggers has not been used for mitigating macroscopic quantities such as traffic delays before.

The remainder of this paper is organized into six sections. Section 2 section briefly discusses prior research related to connected vehicles, V2X communication, and the impact of individual vehicles on traffic flow. Section 3 defines a traffic macrostate that the connected vehicles may seek to impact. Section 4 introduces the notion of zones of influence and event horizon via a thought experiment. Section 5 describes the problem setup using connected vehicles and a fixed freeway bottleneck with emphasis on traffic flow theory and the fundamental diagram of traffic flow. In this section, we also develop an analytical solution for the event horizon and the spatial extent of the ZOI with respect to a specific traffic flow macrostate, building upon and extending the authors’ prior works [12–14] The notation used for the analytical solution can be found in Table 1. Section 6 discusses the various insights that can be gained from these analyses. Finally, Section 7 provides a summary of results and concluding remarks.

**Table 1:** Notation.

*N*	total number of vehicles
*x*_*i*_(*t*)	position of vehicle *i* at time *t* (km)
*v*_*i*_(*t*)	speed of vehicle *i* at time *t* (hr)
Φ(*t*)	traffic macrostate at time *t*
*k*_*Z*_	vehicular density for a generic traffic state *Z* (veh/km)
*q*_*Z*_	traffic flow rate for a generic traffic state *Z* (veh/hr)
*v*_*Z*_	speed of a vehicle within in generic traffic state *Z* (km/hr)
*v*_*YZ*_	speed associated with interface between generic traffic states *Y* and *Z* (km/hr)
$d_{i}^{t}$	delay experienced by *i*^th^ vehicle up to time *t* (veh-hrs.)
$D^{t}$	cumulative delay experienced by all delayed vehicles up to time *t* (veh-hrs.)
$D^{\infty }$	total cumulative delay experienced by all delayed vehicles for the duration of activated bottleneck (veh-hrs.)
*ξ*_*N*_	location of null horizon (km)
*ξ*_*H*_	location of event horizon (km)

## 2 Literature review

In this section, we will discuss existing work in the areas of connected vehicle communications within the context of influencing traffic flow characteristics. A review of the literature indicates that prior works have separately made significant inroads into assessing the effectiveness and relevance of V2X communication networks, as well as in determining the impact of connected autonomous vehicles on traffic flow. However, to the authors’ knowledge, finding analytical solutions for *where* and *to which* Connected Vehicles (CVs) relevant information should be delivered so as to influence macroscopic traffic states remains an open challenge in the research community.

### 2.1 Information relevance and effective V2X communication

For V2X communication to be effective, communication algorithms for Vehicular Ad-hoc Networks (VANETs) should be designed to deliver only relevant information to the appropriate vehicles without unnecessary bandwidth utilization. Prior works have developed measures of information utility [15], analyzed the transmission delays associated with different network topologies [16, 17], and examined the role of communication protocols in reliable and continuous dissemination of information across a vehicular ad-hoc network [18, 19].

With regards to the notion of disseminating information to spatial locations where it is relevant, the research community has worked towards developing the concept of zones of relevance (ZOR) and zones of message forwarding (ZOF), which represent geographical spreads of importance [20, 21]. The zone of relevance has been variously defined as the region where information should be communicated “so that the following vehicle theoretically has the chance to react in time” [22] or a “set of geographic criteria a node must satisfy in order for the geocast message to be relevant to that node” [23]. Prior works have developed methodologies to leverage the zone of relevance and disseminate messages among highly mobile hosts using V2X communication [24].

These protocols were developed to address the broadcast storm problem, which pertains to the bandwidth limitations encountered during attempted information dissemination to all vehicles in a geographical area [25]. Specifically, these concepts have been leveraged to design communication protocols such as geo-casting, which enable transmission of information to a specific geographical areas that are ‘relevant’. For example, event-triggered frameworks have been designed to deliver notifications containing “information related to an event that has potential impact on road safety and traffic condition(s)” [26]. Similarly, context-aware algorithms have been created to select intermediate nodes for message delivery according to various criteria including message importance and current locations of the vehicles [27]. More recent works have sought to develop methods that can send relevant notifications, such as by using vehicular density-based metrics to inform vehicles that may pass through an accident zone [28, 29], or by using probabilistic prediction-based messages for re-routing vehicles based on potential encounters with traffic events [30]. An excellent overview of the topics related to ZOR and geo-casting protocols can be found in [31].

While the aforementioned research is extremely important from an implementation viewpoint, a significant portion such works rely either on heuristics or numerical simulations to identify the zones of relevance. Further, the ZOR literature primarily examines this problem from a communication protocol perspective rather than from the perspective of how this communication can *influence* traffic flow or mitigate congestion. Our work goes beyond the notion of zones of relevance by analytically determining the geographical extent over which a vehicle can influence macroscopic traffic properties—a concept we refer to as *zones of influence* of connected vehicles. To the authors’ knowledge, analytical expressions for determining such zones of influence have not been presented before.

### 2.2 Influencing traffic flow with connected vehicles

Separately, the research community has been actively studying the effects of Connected Vehicles on traffic flow characteristics. For example, studies have examined the effect of CVs on traffic flow in diverse scenarios including signalized intersections [32], bottlenecks [8], highway congestion [33], and urban road networks [34]. Similarly, other research works have studied the impacts on traffic flow arising from distinct implementation schemes including experimental platoons [35], cooperative highway traffic [36], and in mixed traffic flows [37, 38], among others. In the current context, another interesting aspect that influences traffic flow is communication of information to drivers via on-board units which can cause associated changes to driving behaviors such as distracted driving [39, 40]. Overall, one common theme across these research works has been to examine how Connected Vehicles that increase the efficiency of the transportation system through various mechanisms. We now discuss some of these mechanisms such as jam absorption, variable speed limits, and route choice selection, and highlight the differences with respect to the presented concepts of zones of influence and event horizons.

#### 2.2.1 Jam absorption and driver behavior effects

Jam absorption refers to the mechanism by which a single vehicle can slow down to avoid a downstream jam, and in the process help mitigate congestion. Roughly speaking, jam absorption is achieved by ‘slow-in’ and ‘fast-out’ driving behavior, which is characterized by an upstream vehicle slowing down upon noticing a downstream jam, traveling at lower than average traffic speed, and accelerating quickly as the downstream jam begins to dissipate [41]. The role of car-following behavior in the absorption of a traffic jams has been studied both in simulation [42] and experiments [43]. These works have demonstrated that the jam absorption technique can actually help mitigate congestion, provided that an appropriate control policy is applied by the ‘absorbing’ vehicle. Additional works using different modeling techniques (such as Newell’s car-following theory [44] and Totally Asymmetric Simple Exclusion Process (TASEP) model [45]) have examined how controlling the velocity of the ‘absorbing’ vehicle can mitigate congestion near bottlenecks [46]. These concepts have been further advanced in specific application scenarios, e.g., to study fuel consumption reduction [47] and dissipation of stop-and-go waves [48]. More recent works have examined the effects of jam absorption driving algorithms on congestion in various scenarios such as near sags or traffic bottlenecks [49–51]. However, many of these methods rely on numerical simulations to understand the effects on traffic flow. This presents an opportunity to find *analytical, closed-form* approaches that can help provide deeper insights by studying how traffic flow parameters affect congestion and other macroscopic traffic characteristics.

There has also been significant recent empirical research on the effects of driving behavior on traffic flow and macroscopic traffic flow characteristics. Specifically, experimental studies have demonstrated that low penetration rate of autonomous vehicles (AVs) can result in lower driving volatility, higher flow rates, and enhanced oscillation dissipation capabilities [52–54], whereas higher penetration rates do not appear to provide additional benefits. From the perspective of driving algorithms such as adaptive cruise control, field experiments suggest that human drivers are unable to distinguish between human-driven and algorithm-driven vehicles, indicating that current driver assist algorithms may not be able to impact traffic flow [55]. On the other hand, another study indicates that driver assist algorithms have increased response time as compared to human drivers, with potential negative effects on traffic flow [56]. It should also be noted that the macroscopic traffic flow behaviors could change based on the penetration rate of vehicles with traffic flow-focused algorithm or control policy design. For example, analyses based on experimental data offers insight into how the fundamental diagram of traffic flow could change based on various levels of penetration of AVs, with potential higher flow rates with increased penetration rate [29, 57]. However, there seems to be a lack of consensus on the effects of connected autonomous vehicles (CAVs) on traffic flow [58]. Moreover, the studies are inherently limited by the driver assist algorithms *already deployed* by manufacturers, which are primarily designed keeping passenger comfort in mind. It may be instructive to find analytical solutions to understanding macroscopic traffic flow effects, so that the experimental results can be compared against *ideal behaviors*. This approach could help determine potential reasons, methods, and driver assist mechanisms for minimizing deviations from idealized solutions.

#### 2.2.2 Variable speed limits and route choice selection

Similarly, variable speed limits (VSL), variable message signs (VMS), speed harmonization, and route choice selection algorithms are some mechanisms used for mitigating congestion, whose utility and efficacy can be significantly increased with the use of Connected Vehicles. One of the seminal works in this area has been the development of the SPECIALIST algorithm [59]. The algorithm is designed to detect congestion, assess whether application of a lower speed limit in the upstream traffic flow will alleviate congestion, and if so, apply the appropriate control scheme via variable speed limits in the upstream region—thus dissipating shockwaves in traffic flow. However, the algorithm does not explicitly define zones where information dissemination is relevant. though some works extend the SPECIALIST algorithm to V2X scenarios [60]. Similarly, significant research efforts have been directed towards speed harmonization, which uses variable speed limits as a means to actively influence aggregate traffic flow dynamics [8, 61]. Studies have shown that variable message signs (VMS) may be used to display variable speed limits and effectively influence aggregate traffic flow [62].

Recently, there has been increasing interest to understand how to identify optimal locations where VSL approaches may be effective [63], and to examine the effects of VSL zone locations in an urban traffic flow context [64]. However, to our knowledge, the analysis in these works has not yet been extended to connected vehicles. Other research has begun to examine the effects of variable speed limits for Connected Vehicles. For example, studies indicate that speed harmonization may be achieved by connected autonomous vehicles, resulting in higher throughput, lower fuel consumption and emissions, and fewer delays [8, 65, 66]. Specifically, the works of Han et al. share some similarities with the presented work in terms of demonstrating the effects of CV speed control strategies on minimizing delay [8]. While this study seeks to determine the optimal location where activating the CV control strategy has maximum impact, our work provides a more general approach that can not only identify the optimal locations, but also analytically determine the entire spatial range where the disseminated information could generate any amount of positive impacts.

The literature shows that a growing body of methods and results have used heuristics or simulations to determine zones of relevance, and numerical traffic simulations to examine the effects of connected vehicles on traffic flows and congestion mitigation. However, an important question relevant to both VMS deployment and connected vehicles remains unanswered: *where are the most impactful locations to disseminate information for connected vehicles in order to change traffic flow outcomes*? The next section uses a thought experiment to introduce the novel concepts of zones of influence, and event and null horizons. These notions form the first step towards identifying closed-form, analytical solutions for spatial regions, within which information dissemination coupled with specific driving algorithms of Connected Vehicles can positively impact traffic flow.

## 3 Defining a traffic macrostate

To describe zones of influence and associated horizons, we first re-emphasize the notion of a macroscopic state (or ‘macrostate’), which is representative of an overall traffic behavior that one wishes to control. First, the positions and velocities of all the vehicles in the traffic system together are said to define the **microstate** of the system. For example, if the traffic system contains *N* vehicles, then the microstate at a given time instant *t* may be given by $\text{x}(t)=[x_{1}(t),v_{1}(t),x_{2}(t),...,x_{N}(t),v_{N}(t){]}^{⊤}\in {\mathbb{R}}^{2N}$, where *x*_*i*_(*t*) and *v*_*i*_(*t*) represent the position and speed of the *i*th vehicle at time *t*, respectively.

Next, the **macrostate** (Φ(*t*)) is defined as a coarse-scale representation of the large-scale traffic system, i.e. it contains less information about the system than the microstate, but may be better suited to describe the relevant macroscopic-scale dynamics. For example, vehicular density (*k*(*t*)) and flow rate (*q*(*t*)) at time instant *t* represent a *potential* macrostate description of the traffic system, such that $\Phi (t)=[q(t),k(t){]}^{⊤}\in {\mathbb{R}}^{2}$ [67]. There maybe several functional mappings that relate the high-dimensional microstate **x**(*t*) to different low-dimensional macrostates Φ(*t*), at various user-specified spatial scales [68, 69]. Practitioners and researchers may choose to work with such alternative macroscopic variables of interest, e.g., density, net emissions, or total fuel consumption, to name a few. These alternative macrostate definitions may subsequently yield zones of influence of Connected Vehicles and event horizons that differ from those identified for the macrostate proposed in Eq (3). This represents an opportunity to examine traffic flow dynamics from multiple perspectives. Identifying the appropriate macrostate that optimally captures the relevant system dynamics is an open area of research [70–74].

In the authors’ previous work, the macrostate was defined as the number of queued vehicles at a fixed bottleneck on a single-lane freeway [13]. In this work, we use a macrostate of more practical interest—the total vehicular delay D experienced at the fixed bottleneck. Mathematically, the delay $d_{i}^{t}$ experienced by any individual vehicle *i* up to time *t* due to queue formation at the bottleneck may be expressed as:

$$\begin{array}{c} d_{i}^{t}=\{\begin{array}{cc} t-t_{va}^{i}, & t_{va}^{i}\leq t\leq t_{d}^{i}\\ t_{d}^{i}-t_{va}^{i}, & t\geq t_{d}^{i} \end{array} \end{array}$$ (1)

where *t* represents the current time, $t_{va}^{i}(=x_{i}(0)/v_{f})$ represents the virtual arrival time for vehicle *i* at the bottleneck, and $t_{d}^{i}$ represents the departure time for vehicle *i* from the bottleneck. Alternatively, the delay experienced by an individual vehicle up to time *t* may be written as:

$$\begin{array}{c} d_{i}^{t}=\text{min}\left\{t,t_{d}^{i}\right\}-t_{va}^{i} \end{array}$$ (2)

We now define the macrostate as the *aggregate* vehicular delay $D^{t}$ across all affected vehicles, up to time *t* as follows:

$$\begin{array}{c} \Phi \left(t\right)=D^{t}=\sum_{i\in A}d_{i}^{t} \end{array}$$ (3)

where A represents the set of all vehicles that have been delayed due to the activation of the fixed bottleneck. We define the final value of the macrostate (i.e. when the bottleneck becomes inactive again) as the *total* vehicular delay $D^{\infty }=max\;\{D^{t}\}$. The authors chose to use the total vehicular delay at a fixed freeway bottleneck as the macrostate primarily to: (a) focus on the novel concepts of zones of influence and event horizons, (b) obtain an analytical closed-form solution for these concepts, and (c) avoid potential distractions arising from the computational complexity associated with other choices of macrostate (such as net emissions). The evaluation of $D^{\infty }$ is greatly simplified if we assume that the fixed bottleneck is activated only once. We will relax this assumption in our future work.

## 4 Zones of influence and event horizons

In this section we introduce the concept of zones of influence (ZOI) via a thought experiment, in order to provide an intuitive understanding of the zone of influence. The presence of these zones of influence may not be readily evident to drivers, since the zones span spatial scales that may be different from what the driver perceives, but driving behavior can have an impact on them. For example, a driver’s response to a traffic incident report (received via navigation device, radio, or roadside signals) may be contingent on driver characteristics (alertness or risk-aversion), intensity of the incident, and importantly, where and when the information was received [75, 76]. Risk-averse or alert drivers may respond by slowing down *sooner or further upstream* of the incident, but the incident may have been resolved long before they reach the location. On the other hand, drivers that are risk-tolerant or less alert, may respond *too late or too close* to the incident and further exacerbate the effects of the traffic incident. Within these extremes is a zone where a driver’s slow-down response can appropriately reduce the flow of incoming traffic and more effectively mitigate congestion delays.

**Fig 1:**
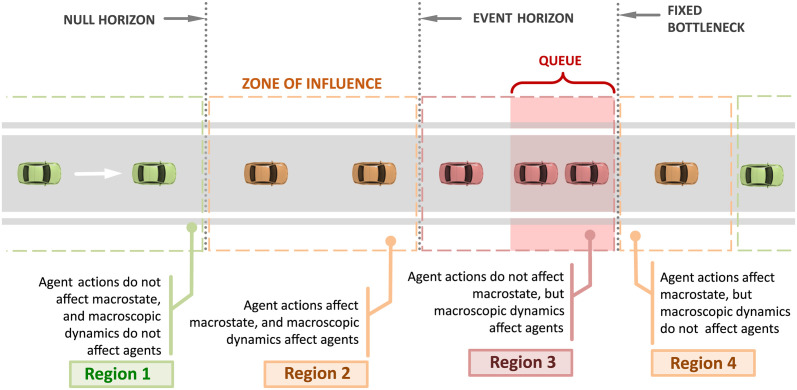
Thought experiment. The concept of zones of influence of Connected Vehicles (CVs) and event horizons in freeway traffic can be undrestood using a thought experiment. White arrow indicates direction of travel from west (left) to east (right). The four regions demarcate the zones where CVs have different impacts on traffic flow (i.e. the macrostate) (Reproduced from [13]).

Fig 1 represents a single-lane roadway with a fixed bottleneck. Vehicles move from left to right, no passing is allowed, and the speed limit is the free flow velocity, *v*_*F*_. We assume that the fixed freeway bottleneck is activated at time *t* = 0 hr. As a consequence, a congested state appears immediately upstream of the bottleneck, and the vehicles in this state travel at a velocity *v*_*H*_ (< *v*_*F*_). As a result of the bottleneck activation, individual vehicles will experience delay $d_{i}^{t}$, the macrostate (i.e. *aggregate* vehicular delay) will be $D^{t}$, and the final value of the macrostate will be the *total* vehicular delay till the bottleneck become inactive again, will be denoted by $D^{\infty }$. In the scenario where Connected Vehicles are absent, the total vehicular delay will be denoted as $D_{0}^{\infty }$.

The final value of the macrostate $\left(D^{\infty }\right)$ may be influenced with the help of Connected Vehicles. In this scenario, we denote the macrostate as $D_{1}^{\infty }$. Ideally, we would like $D_{1}^{\infty }=D_{des}^{\infty }<D_{0}^{\infty }$, where $D_{des}^{\infty }$ represents the desired or acceptable value of total vehicular delay. We assume that the only admissible control policy (or driver algorithm) for a Connected Vehicle is to decelerate quickly to a predefined velocity *v*_*E*_ (> *v*_*H*_) upon receiving information of a downstream jam, and accelerate to free flow speed at a later point in time. This driving algorithm is similar to prior works on jam absorption driving [41]. Now, we consider the impact that a Connected Vehicle receiving information about downstream traffic conditions could have on the final value of the macrostate $D^{\infty }$, for each of the four regions in Fig 1.

**Region 1:** In this region, a Connected Vehicle is situated far upstream of the fixed freeway bottleneck. Irrespective of the congestion information transmitted to this CV, or its potential actions (such as slowing down to speed *v*_*E*_), the congested state is likely to have dissipated by the time the Connected Vehicle reaches the bottleneck. Thus, the CV’s actions are unlikely to positively impact the final value of the macrostate $D^{\infty }$, i.e. $D_{1}^{\infty }\neq D_{des}^{\infty }$ and $D_{1}^{\infty }>D_{0}^{\infty }$. Region 1 is said to extend up to the *null horizon*, which represents a location on the roadway, upstream of which (a) admissible driving algorithms of the CV, and (b) information transmission to the CV, have no positive effect on the delay macrostate $D^{\infty }$.

**Region 2:** In this region, a Connected Vehicle is situated upstream of the queue at the fixed bottleneck, but *downstream* of the null horizon. If the CV chooses to modify its driving behavior (e.g. by slowing down to speed *v*_*E*_ > *v*_*H*_), it would limit the number and rate at which vehicles enter the congested state at the bottleneck. This behavior will reduce total vehicular delay, so that $D_{1}^{\infty }=D_{des}^{\infty }<D_{0}^{\infty }$.

**Region 3:** In Region 3, even if the CV at this location receives information about downstream congestion and takes admissible mitigating actions, it cannot avoid entering the queue or jam as it is already too close to the congested state. Thus, the CV and its following vehicles will continue to join the congested state and experience the same delays as they would have in the absence of the CV. Connected Vehicles in this region have no positive influence on the macrostate, i.e $D_{1}^{\infty }=D_{0}^{\infty }>D_{des}^{\infty }$.

The boundary between Regions 2 and 3 demarcates the roadway into locations where Connected Vehicles can influence the macrostate (Region 2) and locations where they cannot (Region 3), and is referred to as the *event horizon*. Information transmitted to locations upstream of the event horizon may be useful for Connected Vehicles to positively affect the delay macrostate. The event horizon, along with the null horizon, marks the spatial extent of the largest ZOI.

**Region 4:** Now, consider a vehicle at the front of the queue in Region 3 that exits the jam and enters Region 4. In doing so, the vehicle may choose to move at a velocity *v*_*E*_ (<*v*_*F*_), which would incur additional delays for following vehicles. Such behavior is not influential in the sense that a vehicle in this region cannot drive the delay macrostate $D_{1}^{\infty }$ to the pre-determined *improved* state $D_{des}^{\infty }<D_{0}^{\infty }$. However, CV actions in Region 4 can lead to increase in total vehicular delay such that $D_{1}^{\infty }>D_{0}^{\infty }$, which is a negative outcome to be avoided, and may also be of interest. The next section analytically predicts and quantifies the zones of influence and event horizons in the context of mitigating travel delays encountered due to capacity drop at fixed freeway bottlenecks.

## 5 Analytical prediction of zones of influence of connected vehicles

**Fig 2:**
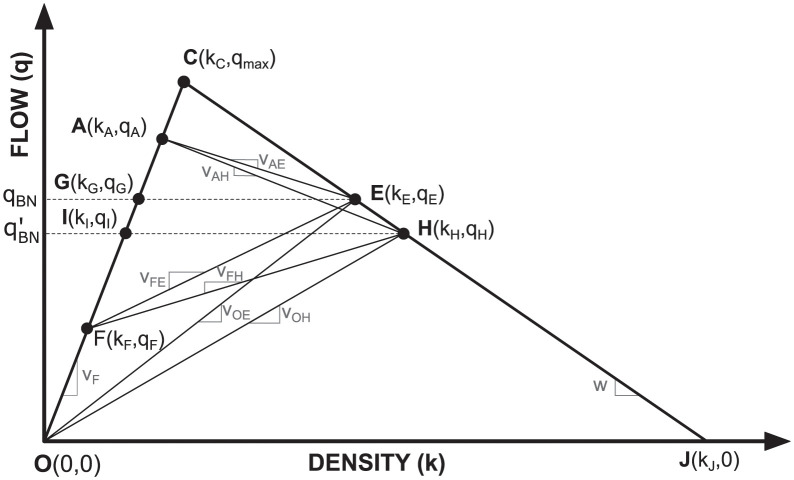
Fundamental diagram of traffic flow denoting states with capacity drop at fixed freeway bottleneck. When the bottleneck is inactive, states *A* and *G* represent the prevalent traffic states upstream and downstream of the bottleneck, respectively. State *E* denotes congested traffic state the bottleneck in absence of capacity drop. States *I* and *H* respectively denote free flow and congested traffic flow states in the presence of capacity drop. Slopes indicate speeds at the state interfaces, as shown in Fig 3.

An analytical example is used for the remainder of the paper to demonstrate how the concept of zones of influence can provide insights into traffic management. We use the same scenario as discussed in the section titled “Zones of Influence and Event Horizons”. Here, we analytically quantify the impact of Connected Vehicles on the delay macrostate *D*^∞^ using an admissible control policy. The approach assumes a triangular relationship between flow and density (Fig 2). We use standard results of the Lighthill-Whitham-Richards (LWR) model, additionally inheriting its assumptions and limitations such as instantaneous changes in vehicle speed. Time-space diagrams are drawn accordingly and can be used to identify the *change* in total vehicular delay (ΔD) due to the actions of CVs, i.e. $\Delta D=D_{1}^{\infty }-D_{0}^{\infty }$. For the remainder of the paper, we drop the ∞ superscript, with the understanding that the symbols $D_{0}$ and $D_{1}$ represent total vehicular delay in the absence and presence of CVs, respectively. Our analysis is performed using representative traffic flow parameters listed in Table 2, unless mentioned otherwise. To facilitate the analytical development of the notion of the ZOI, our subsequent analysis makes a few assumptions, some stronger than others. All of these assumptions have been included at the relevant locations, and collated in Section 6.1.

**Table 2:** Representative traffic flow parameters used in analysis.

Traffic flow parameter	Symbol	Value
Maximum flow	*q*_max_ = *q*_C_	1800 veh/hr
Upstream flow of state A (high flow)	*q*_A_ (= 0.9 *q*_C_)	1620 veh/hr
Upstream flow of state F (low flow)	*q*_F_ (= 0.2975 *q*_C_)	535.5 veh/hr
Rated bottleneck flow	*q*_BN_ = *q*_G_ (= 0.5 *q*_C_)	900 veh/hr
Reduced capacity of bottleneck	*q*′_BN_ = *q*_I_ (= 0.85 *q*_G_)	765 veh/hr
CV1 position (distance to bottleneck at t = 0 hr)	*x*_1_	5 km
Beginning location of low-flow state F (at t = 0 hr)	*x*_F_	20 km
Free flow velocity	*v*_F_	90 km/hr
Jam density	*k*_J_	110 veh/km

**Fig 3:**
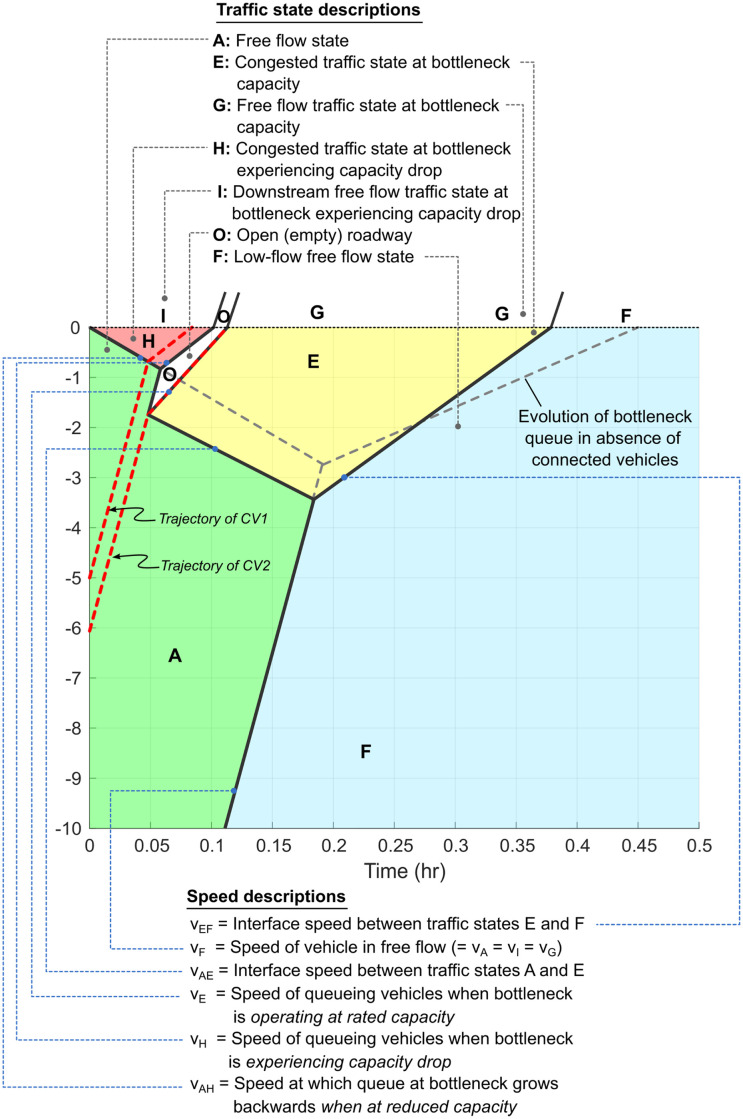
Description of traffic states and interface speeds in the time-space diagram. Dashed grey lines indicate the queue evolution at the fixed freeway bottleneck in the absence of corrective, mitigating actions by the connected vehicles. Connected vehicles trajectories are also included. Please note that this represents a single lane traffic flow scenario, so no lane changes are possible.

In Fig 3, a queue forms at the fixed bottleneck (located at *x*_0_ = 0 km) and vehicles upstream of the queue approach it at free flow velocity (*v*_*F*_). In this figure, at time *t* = 0 hr., the first Connected Vehicle CV1 is at *x*_1_ = 5 km, the second Connected Vehicle CV2 is at *x*_2_ = 6 km, and the nominal position of the low-flow traffic state *F* is located at *x*_*F*_ = 20 km. The bottleneck traffic states associated with capacity drop, i.e. *I* (or *H*) are assumed to incur a 15% drop in flow in comparison to the rated bottleneck capacities given by states *G* (or *E*), i.e. *q*_*I*_ = 0.85*q*_*G*_.

### 5.1 Impact of connected vehicles on total vehicular delay

**Fig 4:**
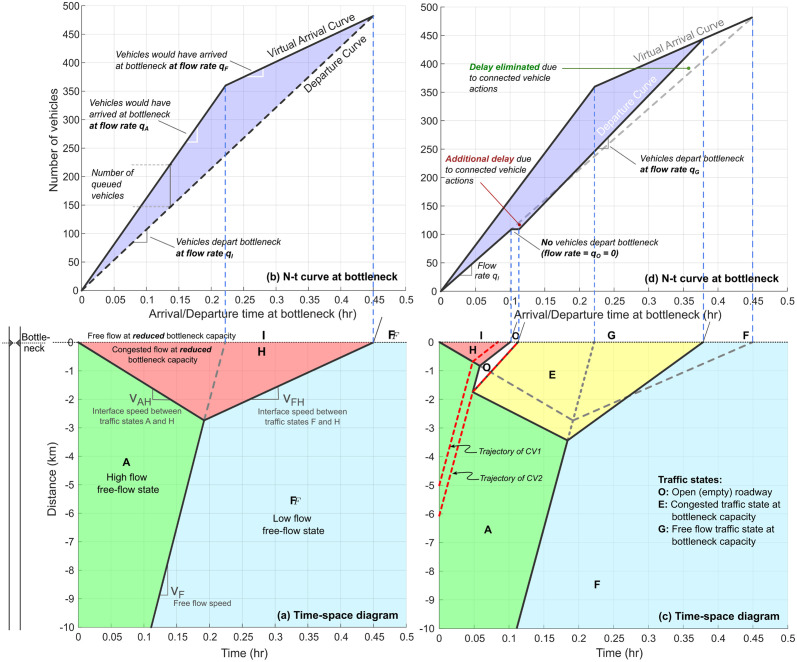
Fixed freeway bottleneck at *x* = 0 km with capacity drop initiated at *t* = 0 hr. Parameter values used to generate plots are included in Table 2. **(a)** Time-space diagram for baseline case without connected vehicles. **(b)** *N* − *t* curve for baseline case with no CVs. **(c)** Alternative traffic evolution when connected vehicles CV1 and CV2 use their communication and event-triggered control policies. **(d)** *N* − *t* curve in the presence of CVs ($D_{0}$).

We now introduce the methodology of evaluating ΔD, i.e. the difference in total vehicular delay in the presence and absence of CVs. The baseline scenario (Fig 4(a) and 4(b)), shows the evolution of traffic flow in the absence of any connected vehicles. The bottleneck initiates at *x* = 0 km and time *t* = 0 hr, and immediately experiences a capacity drop to state *H*. In absence of any intervention by CVs, this state *H* persists until the appearance of the low-flow upstream traffic state *F* causes the queue at the bottleneck to dissipate. The the total vehicular delay $D_{0}$ is calculated using the purple shaded region between the virtual arrival and departure curves of the *N* − *t* curve, as shown in Fig 4(b).

To simplify the analysis, we assume that only two connected vehicles (CV1 and CV2) are present in the traffic flow upstream of the bottleneck, and that these connected vehicles possess a specific event-triggered control policy. For example, in reference to Fig 4(c), when CV1 reaches the queue at around *t* = 0.05 hr., it sends an alert signal to upstream connected vehicles indicating the presence of a queued state. The second Connected Vehicle (CV2) is situated upstream and is assumed to receive this signal instantaneously. The event of receiving this information triggers a control action in CV2, which reduces its speed to *v*_*E*_ (< *v*_*F*_), where *v*_*E*_ denotes the congested flow velocity associated with rated bottleneck capacity state *E*. Connected Vehicle CV2 maintains speed *v*_*E*_ till it crosses the bottleneck at *t* ≈ 0.1 hr, at which point it transitions to free flow speed in traffic state *G*, which corresponds to the rated bottleneck flow. The traffic flow evolution resulting from this event-triggered control policy is shown in the time-space diagram in Fig 4(c). Connected Vehicle trajectories are denoted by dashed red lines in this diagram. Dashed gray lines represent the evolution of the bottleneck states in the absence of Connected Vehicles. Since CV2 is the only vehicle that intentionally slows down, the roadway immediately downstream of CV2 exists as empty space in traffic state *O*. Vehicle that are upstream of CV2, transition to state *E*, since passing is not allowed. The delay macrostate $D_{1}$ can be analytically calculated using the modified area between virtual arrival and modified departure curves, as shown in Fig 4(d). We use various initial positions of CV2 (*x*_2_(0)) to analytically quantify the zone of influence.

We present this analysis from the perspective of a pair of CVs, keeping in mind that this work can be extended to multiple CVs and their associated ZOIs. However, it should be noted that, in the presented analysis, the traffic macrostate is influenced by CV2’s event-triggered control policy *alone*. In other words, the event (i.e., the detection that the bottleneck has been activated) could have been observed by any sensing modality such as another CV, roadside unit, or other infrastructure sensing system. As long as the time of detection is known, the ZOI of CV2 can be evaluated based on the relative position from where the event was first detected. The exact mechanism for detecting the activation of the bottleneck and generation of the alert could be application-specific and adapted to whichever traffic scenario is being analyzed, and would not hinder the following analysis.

**Two insights** are immediately evident from Fig 4(c) and 4(d). **First**, from Fig 4(c) it is observed that the bottleneck returns to free flow state earlier, i.e. at around *t* = 0.375 hr instead of around *t* = 0.45 hr which was the baseline scenario (Fig 4(a)). Thus, the inclusion of CV1 and CV2 along with their admissible control policies results in quicker deactivation of the bottleneck. The **second insight** is that while CV2’s event-triggered control policy may add some delay initially, it eliminates a significantly greater quantity of vehicular delay over the duration of the active bottleneck, thus producing a net reduction in total delay. In other words, CV2 is able positively influence the delay macrostate, such that $D_{1}<D_{0}$.

### 5.2 Analytical solution and quantification of zones of influence

S1 and S2 Appendices show how to obtain mathematical expressions for total vehicular delays in the presence and absence of CVs. We use these to determine the locations of the event and null horizons, and consequently, the extent of the ZOI, as shown below.

#### 5.2.1 Analytical expression for the null horizon

S1 Appendix shows the derivation of the expression for the total vehicular delay $D_{0}$ at the fixed freeway bottleneck in the *absence* of mitigating actions by CVs (baseline scenario). In this scenario, the delay $D_{0}$ depends only on the traffic state parameters θ = {*q*_*A*_, *q*_*I*_, *x*_*F*_, *v*_*F*_, *v*_*AH*_, *v*_*FH*_} (defined in Fig 2), and the position *x*_*F*_ of the low-flow free flow state *F* at time *t* = 0. As shown in Eq (7), the expression for $D_{0}$ is given by:

$$\begin{array}{c} D_{0}\left(\theta \right)=\frac{1}{2}\;\left(q_{A}-q_{I}\right)\cdot \{\frac{v_{AH}+v_{FH}}{v_{F}\cdot v_{FH}\cdot \left(v_{AH}+v_{F}\right)}\}\cdot x_{F}^{2}=\lambda_{0}\cdot x_{F}^{2} \end{array}$$ (4)

where the constant $\lambda_{0}=\frac{1}{2}\;(q_{A}-q_{I})\cdot \left\{\frac{v_{AH}+v_{FH}}{v_{F}\cdot v_{FH}\cdot (v_{AH}+v_{F})}\right\}$. Similarly, S2 Appendix shows the derivation of the expression for the total vehicular delay $D_{1}$ in the presence of event-triggered control policies of the CVs. Consequently, in addition to traffic state parameters θ, the delay $D_{1}$ also depends on the positions *x*_1_ and *x*_2_ of the connected vehicles CV1 and CV2, respectively. As shown in Eq (B.18) in S2 Appendix, the expression for $D_{1}$ is given by:

$$\begin{array}{c} D_{1}\left(x_{1},x_{2},\theta^{\prime }\right)=\;\lambda_{1}x_{F}^{2}+\lambda_{2}x_{2}^{2}+\lambda_{3}x_{1}^{2}+\lambda_{4}x_{1}x_{2}+\lambda_{5}x_{F}x_{2}+\lambda_{6}x_{F}x_{1} \end{array}$$ (5)

where θ′ = {θ, *x*_1_, *x*_2_}. The parameters λ_1_ through λ_6_ are defined in Eq (B.18) in S2 Appendix and depend only on traffic states and velocities found in the fundamental diagram of traffic flow as shown in Fig 2.

We quantify the potential positive impact of the second scenario (with CVs)—in the form of reduced total vehicular delay—as compared to the first scenario (without CVs). Specifically, we examine the net change in delay $\Delta D=D_{1}-D_{0}$ using Eqs (4) and (5) as follows:

$$\begin{array}{cc} \Delta D & =\;D_{1}-D_{0}\\ & =\;\left(\lambda_{1}-\lambda_{0}\right)\;x_{F}^{2}+\lambda_{2}x_{2}^{2}+\lambda_{3}x_{1}^{2}+\lambda_{4}x_{1}x_{2}+\lambda_{5}x_{F}x_{2}+\lambda_{6}x_{F}x_{1} \end{array}$$ (6)

If ΔD<0, i.e. $D_{1}<D_{0}$, then the actions of connected vehicles lead to a reduction in total delay experienced by vehicles at the fixed freeway bottleneck—a net positive impact. We now determine the spatial regions where CV2’s control actions generate this positive impact. Assuming that the values of *x*_1_ and *x*_*F*_ are fixed, we re-write the above expression as a function of the position of CV2 (*x*_2_) alone, as follows:

$$\begin{array}{cc} \Delta D & =\;\lambda_{2}x_{2}^{2}+(\lambda_{4}x_{1}+\lambda_{5}x_{F})\;x_{2}+(\left(\lambda_{1}-\lambda_{0}\right)\;x_{F}^{2}+\lambda_{3}x_{1}^{2}+\lambda_{6}x_{F}x_{1})\\ & =\;\Lambda_{2}x_{2}^{2}+\Lambda_{1}x_{2}+\Lambda_{0} \end{array}$$ (7)

where Λ_2_ = λ_2_, Λ_1_ = λ_4_*x*_1_ + λ_5_*x*_*F*_, and $\Lambda_{0}=(\lambda_{1}-\lambda_{0})\;x_{F}^{2}+\lambda_{3}x_{1}^{2}+\lambda_{6}x_{F}x_{1}$.

The null horizon *ξ*_*N*_ is given by the smallest positive root of ΔD=0. Solving for *x*_2_ in Eq (7) we obtain an expression for the null horizon:

$$\begin{array}{c} \xi_{N}=\underset{x_{2}}{\text{arg}\;\text{min}}\;\{x_{2}:\Lambda_{2}x_{2}^{2}+\Lambda_{1}x_{2}+\Lambda_{0}=0,\;\underline{\text{and}}\;x_{1}<x_{2}<x_{F}\;\} \end{array}$$ (8)

It is also instructive to discuss the location of the null horizon in relation to the position of the first connected vehicle CV1, i.e. $\xi_{N}^{\prime }=\xi_{N}-x_{1}$.

#### 5.2.2 Analytical expression for the Event horizon

On the other hand, the mathematical expression for the event horizon is derived using the time instances when the traffic states *O* and *E* first arrive at the fixed freeway bottleneck, as shown in Fig 4. Using the expressions derived in S2 Appendix, the times at which traffic states *O* and *E* first reach the bottleneck are given by *t*_*o*_ and *t*_2_, respectively, and are reproduced here for convenience from Eqs (B.5) and (B.6) in S2 Appendix:

$$\begin{array}{cc} & \;t_{o}=\frac{1}{v_{H}}\cdot (\frac{v_{AH}+v_{H}}{v_{AH}+V_{F}})\cdot x_{2} \end{array}$$ (9)

and,

$$\begin{array}{c} \;t_{2}=\frac{1}{v_{E}}\cdot \{x_{2}-(\frac{v_{F}-v_{E}}{v_{AH}+v_{F}})\cdot x_{1}\} \end{array}$$ (10)

The second connected vehicle (which is also the lead vehicle of traffic state *E*) can positively impact the net vehicular delay at bottleneck only if *t*_2_ > *t*_*o*_ as is evident from Fig 6(a)–6(d), as well as in Fig 12 in S2 Appendix. Consequently, the event horizon is given by equating *t*_2_ to *t*_*o*_, or:

$$\begin{array}{cc} \frac{1}{v_{H}}\cdot (\frac{v_{AH}+v_{H}}{v_{AH}+V_{F}})\cdot x_{2} & =\frac{1}{v_{E}}\cdot \{x_{2}-(\frac{v_{F}-v_{E}}{v_{AH}+v_{F}})\cdot x_{1}\}\\ \text{or},\;\;\{\frac{1}{v_{E}}-\frac{1}{v_{H}}(\frac{v_{AH}+v_{H}}{v_{AH}+V_{F}})\}\cdot x_{2} & =\frac{1}{v_{E}}(\frac{v_{F}-v_{E}}{v_{AH}+v_{F}})\cdot x_{1}\\ \text{or,}\;\;\xi_{E}=x_{2} & =\{\frac{\left(v_{F}-v_{E}\right)\cdot v_{H}}{\left(v_{F}-v_{E}\right)\cdot v_{H}+\left(v_{H}-v_{E}\right)\cdot v_{AH}}\}\cdot x_{1} \end{array}$$ (11)

where *ξ*_*E*_ represents the event horizon boundary, downstream of which CV2’s event-triggered actions have no positive impact on the total vehicular delay at the bottleneck. As before, it is instructive to discuss the position of the event horizon in relation to the position of CV2, i.e. $\xi_{E}^{\prime }=\xi_{E}-x_{1}$:

Further, knowledge of the analytical expressions for the null and event horizons enables us to quantify the extent of the zone of influence as the range (*ξ*_*E*_, *ξ*_*N*_). Within this zone, CV2’s admissible event-triggered control policy can positively impact the traffic macrostate ($D_{1}<D_{0}$, or ΔD<0).

## 6 Results

**Fig 5:**
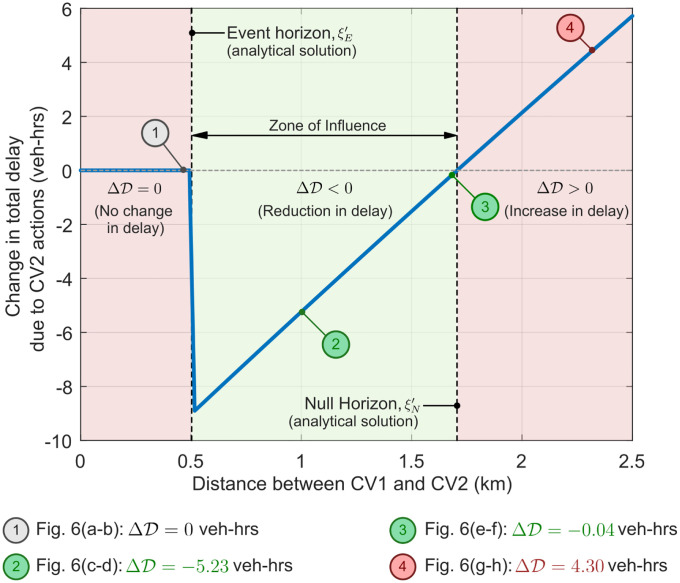
Change in total vehicular delay ΔD as a function of initial separation between connected vehicles CV1 and CV2 (i.e. at time *t* = 0 hr). Analytical solutions for event and null horizons are based on parameters included in Table 2. The region between the event and null horizon is the *zone of influence*, where CV2’s control policy can positively impact traffic flow. Points 1, 2, 3, and 4 represent Rows 1, 2, 3, and 4 in Fig 6, respectively. E.g., Fig 6(a) and 6(b) shows the time-space diagram for Point 1 in Fig 5. Both figures represent the same traffic flow scenario with identical parameters.

In this section, we present additional results for several traffic state-dependent scenarios to make analytical predictions for the zone of influence, and event and null horizons as developed in Section 5.2. Fig 5 shows the change in total vehicular delay (ΔD) at the fixed freeway bottleneck as a function of the position (*x*_2_) of the second connected vehicle CV2. This figure also shows the analytical solutions for the null horizon $\left(\xi_{N}^{\prime }\right)$ and event horizon $\left(\xi_{E}^{\prime }\right)$. The x-axis depicts the relative distance between CV1 and CV2 at time *t* = 0 hr, while the y-axis denotes the macrostate of interest, i.e. the change in total vehicular delay ΔD. Unless otherwise mentioned, all analysis in this work is performed using representative traffic flow parameters listed in Table 2.

It is evident from the Fig 5 that the location of CV2 has a distinct impact on its ability to affect the macrostate ΔD. The zone of influence is given by the region [0.5, 1.7] km in this scenario, where ΔD<0 vehicle-hours (veh-hrs.) Outside this spatial region, the specific event-triggered actions of CV2 either have no effect (ΔD=0 veh-hrs. in region [0, 0.5] *km*) or negatively impact the macrostate, thus increasing the vehicular delay (ΔD>0 veh-hrs. in region [1.7, ∞) km). The event horizon exists at $\xi_{E}^{\prime }=0.5\;km$, and the null horizon exists at $\xi_{N}^{\prime }=1.7\;km$.

**Fig 6:**
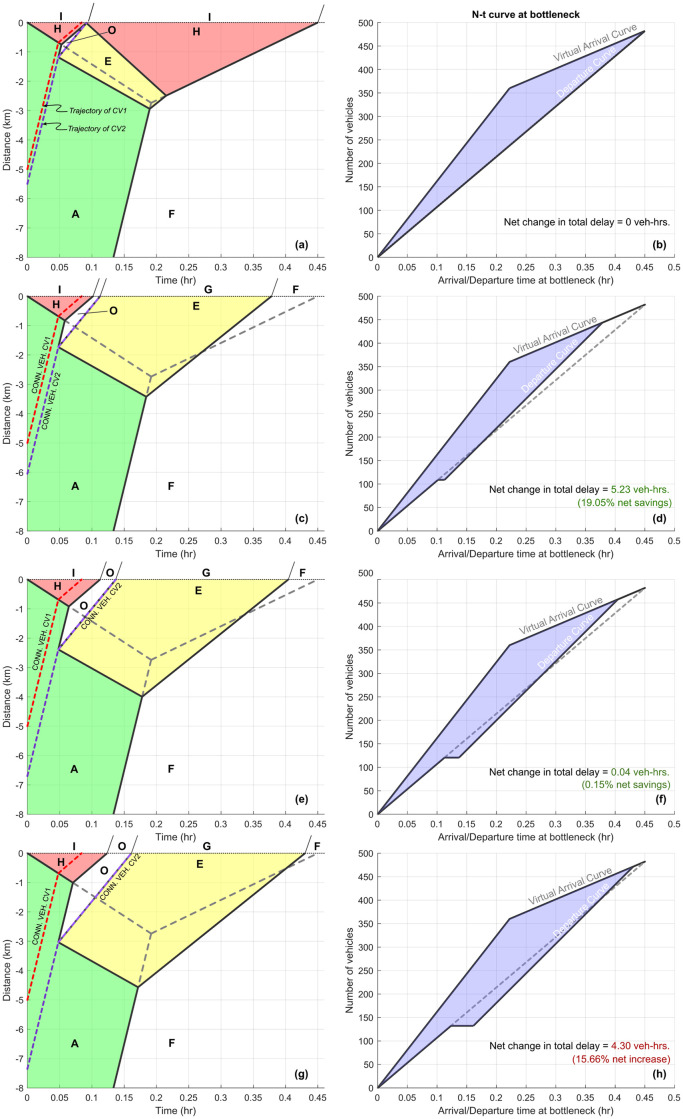
Time-space diagrams and *N* − *t* curves at the bottleneck for various positions of connected vehicle CV2 with respect to CV1. Point 1 in Fig 5 corresponds to Fig 6(a) and 6(b), Point 2 corresponds to Fig 6(c) and 6(d), Point 3 corresponds to Fig 6(e) and 6(f), and Point 4 corresponds to Fig 6(g) and 6(h). Traffic flow parameters are the same as those used in Fig 5.

Fig 6 depicts the time-space diagram and *N* − *t* curves at the bottleneck when CV2 is positioned at different locations with respect to CV1. Fig 6(a) and 6(b) shows that if CV2 is downstream of the event horizon ($\xi_{E}^{\prime }$) (i.e. Region 3), then its control actions produce no change in total vehicular delay at the fixed bottleneck, i.e. ΔD=0 veh-hrs. Point 1 in Fig 5 corresponds to Fig 6(a) and 6(b) Similarly, Fig 6(c) and 6(d) corresponds to Point 2 in Fig 5, and depicts the positive impact of CV2’s actions within its zone of influence (i.e. Region 2). In this scenario, CV2’s actions lead to ΔD<0, with a total delay savings of 5.23 veh-hrs., or 19.05% as compared to baseline scenario with no CV actions. Fig 6(e) and 6(f) corresponds to Point 3 in Fig 5 (still in Region 2), and shows that when CV2 is closer to the null horizon, the net positive impact on the macrostate is reduced. Beyond the null horizon $\xi_{N}^{\prime }$ (i.e. Region 1), CV2’s actions negatively impact the macrostate, as shown in Fig 6(g) and 6(h) corresponding to Point 4 in Fig 5. In this scenario, CV2’s actions produce a net *increase* in total vehicular delay equivalent to 4.30 veh-hrs., or 15.66% as compared to baseline scenario. If CV2 is positioned further upstream and continues to enact this event-triggered control policy, it will negatively impact the delay macrostate with even larger magnitude. It is worth noting that this result is also significant: ‘blind’ adherence to a control policy may lead to undesirable outcomes, further reinforcing the need to study feedback and coupling between the zone of influence *and* control policy decisions made by CVs.

In all the scenarios considered so far, the zone of influence of CV2 is determined for specific traffic states: *A* (state immediately upstream of bottleneck), *G* (state associated with rated bottleneck capacity), and *F* (low-flow free flow state far upstream of the bottleneck), described by parameters in Table 2. However, the analytical solutions developed in Section 5.2 allow us to observe more general patterns in the extent of the zone of influence, and the locations of the event and null horizons, as the variables such as (*k*_*A*_, *q*_*A*_), (*k*_*G*_, *q*_*G*_), and (*k*_*F*_, *q*_*F*_) change. These generalized results are discussed in the next few subsections. However, before proceeding to the results, it is instructive to review the basic assumptions that underlie this work.

### 6.1 Assumptions and caveats

Since the analytical determination of the zones of influence presented here is performed in a highly idealized traffic flow scenario, the authors acknowledge the need to clearly outline the key (and sometimes strong) assumptions. In this subsection, we also discuss how these assumptions can limit the ‘direct’ application of this work to real-world scenarios. The authors would like to emphasize that the discussion in this subsection *does not* diminish the conceptual contribution of our work, but instead provides various guidelines for readers on how its limits can be tested in future research.

- *Single lane assumption:* To facilitate the analytical development of the ZOI, we assume that the traffic flow scenario comprises of a single lane. This assumption implies that lane changing behaviors that are an inherent part of many traffic flow scenarios have not been included in the presented analysis.
  If the analysis were to include multiple lanes and lane changing behaviors, we expect a significant reduction in the ability of a single connected vehicle to impact traffic flow mitigate vehicular delay. We foresee future modifications and expansion of the concept of ZOI that can help address this issue.
- *CV2 control policy:* We have assumed a simple event-triggered control policy to derive the analytical results. Alternative control policies can also be used, and these would likely produce different zones of influence for CV2.
- *Bottleneck activation:* We have assumed that the bottleneck is activated only once at the beginning of the simulation. This assumption enables us to obtain analytical results in our work. For example, a secondary bottleneck activation could occur due to the actions of CV2. Additionally, the activation of the bottleneck could produce vehicular clusters that are not fully captured by the LWR model used in our analysis.
- *Parameters used:* The analysis in this work has been performed using the set of parameters as described in Table 2, where some of the parameters such as *x*_1_, *x*_*F*_, *v*_*F*_, *k*_*J*_ etc. are fixed. While changes to these parameters are not expected to have a qualitative impact, they are bound to have an impact of the quantitative discussions in Sections 6.2–6.5, such as the results included in Table 3.
- *Fundamental diagram:* We assume a triangular fundamental diagram with typical traffic flow parameters. There is a significant body of work associated with the fundamental diagram and its variations, but making this assumption appears to be reasonable in the current context.
- *Instantaneous communication:* We assume that CV2 receives the alert signal from CV1 instantaneously. While this is not strictly true, we do not anticipate that any realistic values of communication delay will impact the presented analysis.
- *Message hopping requirements:* To better understand the minimum penetration rate requirements, we assume that the CVs are uniformly distributed across the roadway. While relaxing the assumption will have some effect, we do not expect it to change the qualitative results.

**Table 3:** Summary of qualitative results obtained from quantifiable analytical expressions of ZOI *using the parameters specified in* Table 2.

Ratio of interest	Values	Average delay reduction	Length of zone of influence (km)	Min. penetration rate
*q*_*G*_/*q*_*A*_	**small** (∼ 0.4)	**large** (∼ 60 veh-hrs)	**large** (∼ 10 km)	**medium** (∼ 3%)
	medium (∼ 0.7)	medium (∼ 10 veh-hrs)	small (∼ 1 km)	large (∼ 4%)
	large (∼ 1.0)	small (∼ 2 veh-hrs)	medium (∼ 5 km)	small (∼ 1%)
*q*_*A*_/*q*_*C*_	small (∼ 0.5)	small (∼ 2 veh-hrs)	small (∼ 0.1 km)	large (∼ 30%)
	**large** (∼ 1.0)	**large** (∼ 16 veh-hrs)	**large** (∼ 1 km)	**small** (∼ 5%)
*q*_*F*_/*q*_*I*_	small (∼ 0.0)	small (∼ 20 veh-hrs)	small (∼ 0.1 km)	large (∼ 6%)
	**large** (∼ 1.0)	**large** (∼ 80 veh-hrs)	**large** (∼ 1 km)	**small** (∼ 2%)

### 6.2 Dependence on rated bottleneck state *G*(*k*_*G*_, *q*_*G*_)

**Fig 7:**
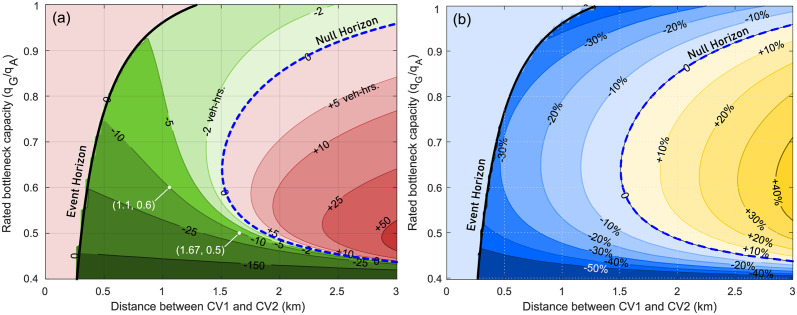
Change in total vehicular delay (veh-hrs.) at the fixed freeway bottleneck as a function of CV1-CV2 separation *and* rated bottleneck capacity. Thick black line denotes the event horizon $\xi_{E}^{\prime }$, while dashed blue line represents the null horizon $\xi_{N}^{\prime }$. The y-axis denotes the rated bottleneck capacity *q*_*G*_ as a fraction of the high-flow upstream traffic flow *q*_*A*_. Contours denote the net change in delay (absolute (a) and percentage (b)) due to the actions taken by CV2 if it were present at the location indicated by the x-axis.

Now, we analytically quantify the dependence of the event horizon, null horizon, and extent of the zone of influence of CV2 on the rated capacity *q*_*G*_ of the fixed freeway bottleneck. Fig 7(a) and 7(b) use the expressions developed in S1 and S2 Appendices to depict the total vehicular delay at the bottleneck as a function of the distance between CV1 and CV2, for a range of rated bottleneck capacities *q*_*G*_ and while the flow *q*_*A*_ of upstream traffic state *A* is held constant. The y-axis denotes the ratio *q*_*G*_/*q*_*A*_, and the contours denote identical changes in the macrostate, i.e. the change in total vehicular delay ΔD is a constant on these contour lines. Based on the problem setup in Section 5, the value of *q*_*G*_ is assumed to be lower-bounded such that *q*_*F*_ ≤ *q*_*I*_ < *q*_*G*_, and upper-bounded by *q*_*A*_, such that *q*_*G*_/*q*_*A*_ ≤ 1. Furthermore, the total vehicular delay is measured in units of veh-hrs. (or percentage change), and darker shades indicated higher magnitudes (or greater percentage change). Thus, dark green (Fig 7(a)) and blue (Fig 7(b)) indicate greatest savings or reduction in total vehicular delay D, whereas dark red and yellow indicates greatest increase in delay D.

As an example in Fig 7(a), when CV2 is located about 1.1 km upstream of CV1 and *q*_*G*_/*q*_*A*_ = 0.6, then the activation of the event-triggered control policy changes the total delay by ΔD≈−10 veh-hrs., i.e. there is a net reduction in total vehicular delay. The same net reduction is achieved when *q*_*G*_/*q*_*A*_ = 0.5 and the CV2 is located about 1.67 km upstream of CV1, indicating that both locations are within the zones of influence. It is also evident from Fig 7 that delay savings are highest for CV actions taken closest to the event horizon, and continue to decrease further away from it.

Several key insights can be gleaned from Fig 7. Importantly, the space between the event and the null horizons represents the zones of influence, where specific event-triggered control policies used by CV2 can positively impact the traffic macrostate. The **first key insight** is that the zones of influence exists for all reasonable values of *q*_*G*_/*q*_*A*_ that we may expect to observe in traffic, indicating its potential utility and relevance in better management of traffic. The **second key insight** is that the actions of connected vehicle CV2 in the region closer to the bottleneck than the event horizon have no impact on the macrostate (total vehicular delay). Thus, in the current context, there may be limited utility in communicating with connected vehicles in this region, except for passing the information to a CV in the zones of influence in a V2V scenario. Furthermore, actions of CV2 in the region farther from the bottleneck than the null horizon are also non-beneficial to the traffic system macrostate. Thus, control actions in this region, even if proven to be beneficial in other locations, are actually detrimental to achieving the desired macrostate objectives. These results illustrate that *communication of relevant information is perhaps best reserved for and delivered only to those vehicles that are within their zones of influence*.

A **third key insight** obtained from Fig 7 by observing that as the ratio *q*_*G*_/*q*_*A*_ increases, i.e. the rated capacity *q*_*G*_ is closer to the fixed upstream flow *q*_*A*_, the *magnitude* of effects of the CV control policies on total vehicular delay are significantly diminished. Observing the top of Fig 7(a) and 7(b), the zones of influence appears to be large due to the widening gap between the event and null horizons, but the net *magnitude* of influence of the CV control actions is negligible (approximately 2 veh-hrs of delay savings). However, the top of Fig 7(b) shows that the *percentage change* in total vehicular delay at high ratios of *q*_*G*_/*q*_*A*_ is still significant (up to 40%) and comparable to other flow ratios, even if the *magnitude* of change seems insignificant. For the **fourth key insight**, we observe the bottom of Fig 7(a) and 7(b) where the ratio *q*_*G*_/*q*_*A*_ is small, or *q*_*I*_ (= 0.85*q*_*G*_) ≈ *q*_*F*_. If the reduced bottleneck capacity *q*_*G*_ is significantly smaller than upstream traffic flow *q*_*A*_, then long delays can be expected at the bottleneck. This is a direct consequence of scenario where the bottleneck operates at reduced capacity for long duration without the potential mitigating actions of connected vehicles. In this scenario, the specific event-triggered control policy of CV2 will positively impact the macrostate, irrespective of how much further upstream of the event horizon CV2 is positioned (a result that can be attributed to the pervasive nature of the bottleneck). Of course, as seen at the bottom of Fig 7(a) and 7(b), the positive impact (magnitude or percentage change) generated due to CV2 diminishes the farther away from the bottleneck it is, with the most significant reduction in total vehicular delay occurring when CV2 is close to the event horizon. Comparing the top and bottom of Fig 7(b), we notice that though the extents of the the zones of influence in both cases are comparable, the net positive effect is much greater for *q*_*G*_ ≪ *q*_*A*_ than for *q*_*G*_ ≈ *q*_*A*_. In other words, a Connected Vehicle has a significantly larger zones of influence when the bottleneck has a relatively low rated capacity.

With knowledge of the rated bottleneck capacity, we can *quantify* the spatial extents over which communication is useful, and in turn determine communication strategies with connected vehicles. Combining analytical predictions of the zone of influence with appropriate knowledge of the upstream traffic flow state *A*, we can leverage the presence of connected vehicles to positively impact traffic flow macrostates. Next, we consider the effects of upstream traffic flow state *A* on the extent of the zone of influence and the positions of the event and null horizons.

### 6.3 Dependence on upstream high-flow traffic state *A*(*k*_*A*_, *q*_*A*_)

**Fig 8:**
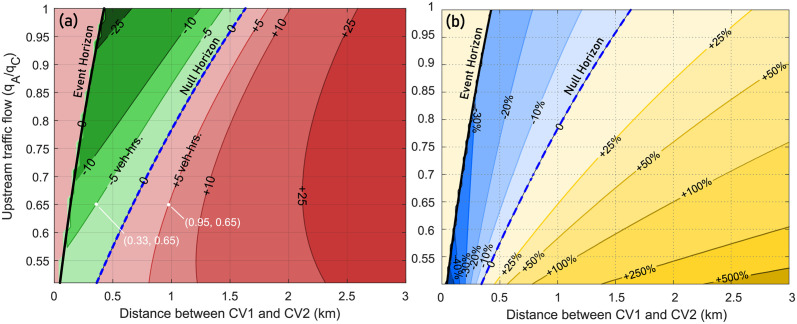
Extent of zone of influence for varying upstream traffic flow state *A*(*k*_*A*_, *q*_*A*_). Contours denote the net *magnitude* change ((a), left), and the net *percentage* change ((b), right) in total vehicular delay at the bottleneck due to the actions taken by CV2 if it were present at the location indicated by the x-axis (in relation to the position of CV1). Thick black line denotes the event horizon $\xi_{E}^{\prime }$, while dashed blue line represents the null horizon $\xi_{N}^{\prime }$.

Similar to the discussion in the previous subsection, here we analyze the impact of various upstream traffic states *A*(*k*_*A*_, *q*_*A*_) on the zones of influence and horizons, with the parameters of the traffic states *C*, *F*, and *G* held constant. Based on the problem setup in Section 5, the value of *q*_*A*_ is assumed to be lower-bounded by *q*_*G*_, and upper-bounded by *q*_*C*_, such that *q*_*A*_/*q*_*C*_ ≤ 1. The expressions in S1 and S2 Appendices are used to obtain the contour plots in Fig 8(a) and 8(b). The y-axis now represents the ratio of the upstream traffic flow *q*_*A*_ to the critical maximum flow *q*_*C*_ (i.e. *q*_*A*_/*q*_*C*_). For example, as shown in Fig 8(a), if the upstream traffic flow is *q*_*A*_/*q*_*C*_ = 0.65, then an event-triggered control action by CV2 positioned about 0.33 km upstream of CV1 leads to a 5 veh-hrs *reduction* in total vehicular delay, a net positive impact on the traffic macrostate. On the other hand, the same event-triggered control action by CV2 if it were positioned at 0.95 km upstream of CV2 would *increase* total vehicular delay by 5 veh-hrs, a net negative impact on the traffic macrostate.

Additional insights based on Fig 8 are now discussed. As before, we see that communication directed towards vehicles downstream of the event horizon does not benefit the traffic macrostate in the current context and scenario. Specific to the parameters associated with traffic state *A*, **the key insight** is that the extent of the zone of influence is larger for higher values of the upstream traffic flow *q*_*A*_, i.e. as *q*_*A*_/*q*_*C*_ → 1. This is intuitive, considering that capacity drop induced by higher upstream traffic flow *q*_*A*_ is likely to produce queues that are larger than in a scenario with lower upstream traffic flow. Thus, due to the potential for larger queue formation, the connected vehicle CV2 can positively impact the traffic macrostate with its event-triggered control policy even if it is present at spatial locations farther upstream of CV1. This also is indicated by the location of the null horizon, which is farther upstream at higher upstream flow rates *q*_*A*_. More importantly, due to the potential for larger queue formation when *q*_*A*_ → *q*_*C*_, CV2’s actions produce significant reduction in vehicular delay.

### 6.4 Dependence on upstream low-flow traffic state *F*(*k*_*F*_, *q*_*F*_)

**Fig 9:**
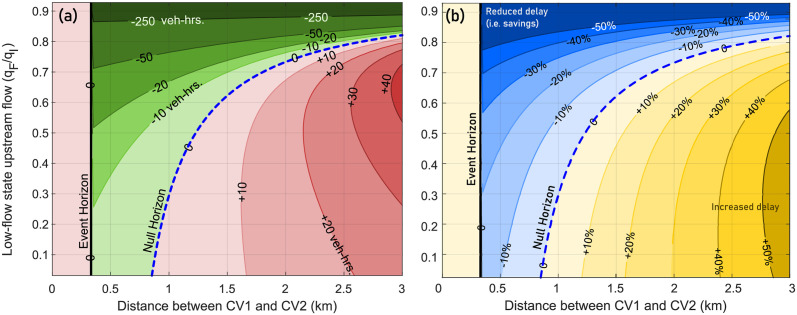
Zone of influence is larger for higher flows associated with low-flow upstream traffic state *F*. Thick black line denotes the event horizon $\xi_{E}^{\prime }$, while dashed blue line represents the null horizon $\xi_{N}^{\prime }$. The y-axis denotes the upstream flow of state *F* (*q*_*F*_) as a fraction of the reduced bottleneck capacity *q*_*I*_. Contours denote the net change in delay due to the actions taken by CV2 if it were present at the location indicated by the x-axis. Additionally, reduced flow *q*_*I*_ is always equal to 0.85 *q*_*G*_, denoting a 15% drop in bottleneck capacity upon initiation.

Similar to the traffic flow *q*_*A*_ of the upstream traffic state *A*, it is also expected that the flow *q*_*F*_ of the low-flow upstream state *F* will also impact the extent of the zone of influence. The analysis of the effects of the low flow *q*_*F*_ is discussed with reference to Fig 9, where the y-axis now represents the ratio of the low-flow upstream traffic flow *q*_*F*_ to the reduced bottleneck capacity *q*_*I*_ (i.e. *q*_*F*_/*q*_*I*_). In relation to the problem setup in Section 5, the value of *q*_*F*_ is assumed to be lower-bounded by 0, and upper-bounded by *q*_*I*_, such that 0 ≤ *q*_*F*_/*q*_*I*_ ≤ 1. Fig (9) shows the change in total vehicular delay due to the actions of CV2.

Two insights stand out distinctly in Fig 9 when compared against Fig 8. **The first insight** is that the location of the event horizon remains unchanged for any value of the upstream flow *q*_*F*_. In other words, the traffic state *F* far upstream of the bottleneck has no relation to the limiting extent (i.e. event horizon) of where CV2’s actions can positively impact the traffic macrostate. The **second insight** is that the location of the null horizon changes significantly for varying values of the upstream traffic flow *q*_*F*_. Specifically, for higher values of upstream traffic flow given by *q*_*F*_/*q*_*I*_ → 1, formation of larger bottleneck queues is observed. As seen at the top of Fig 9(a) and 9(b), CV2’s actions result in significant reductions in total vehicular delay in both magnitude *and* percentage. This can be understood as follows: In this scenario CV2 has many opportunities and upstream locations where it can activate the event-triggered control policy to positively impact the traffic macrostate. Moreover, as *q*_*F*_ → *q*_*I*_ the downstream traffic state begins to more closely resemble the rated capacity state of the bottleneck, so any mitigating action is bound to have a major impact. Indeed, at higher flow rates, not only is the null horizon farther upstream, the net positive impact is also several orders of magnitude higher than for similar situations for lower values of *q*_*F*_, which is a direct result of many more vehicles being directly influenced by the actions of CV2.

### 6.5 Message hopping and minimum penetration rate requirements

On the basis of these results, we can obtain some additional insights into the minimum message hopping and penetration rate requirements to successfully mitigate delays caused as a result of capacity drop near fixed freeway bottlenecks.

#### 6.5.1 Message hopping requirements

As discussed in Section Analytical Solution and Quantification of Zones of Influence, delay mitigation is only possible if CVs located close to the bottleneck (downstream of the event horizon) are able to communicate with at least one connected vehicle (such as CV2) located in the zone of influence. To understand minimum message hopping requirements, we determine the number of hops required for a Connected Vehicle entering a queue (e.g., CV1) to communicate a message to a Connected Vehicle (e.g., CV2) that is situated just upstream of the event horizon. Using standard approximations of communication range to be 300 m, we can evaluate the minimum number of message hops between CV1 and CV2 that would enable delay mitigation [77]. For example, as seen in Fig 7(b), if the rated bottleneck capacity *q*_*G*_ is close to the known upstream traffic flow *q*_*A*_ with all other parameters held constant (i.e. *q*_*G*_/*q*_*A*_ → 1), the event horizon is significantly further upstream as compared to lower values of *q*_*G*_. Consequently, in order to influence the traffic macrostate, connected vehicles CV1 and CV2 would have to communicate over a range of approximately 1200 m, requiring about three message hops. However, since *q*_*G*_ ≈ *q*_*A*_ in this scenario, the net positive impact would remain limited. On the other hand, for example, when *q*_*G*_/*q*_*A*_ ≈ 0.4, the event horizon *ξ*_*E*_ ≈ 300 m, so delay may be mitigated by direct communication between CV1 and CV2. Similarly, in the scenarios discussed in Fig 8(a) and 8(b), For example, when *q*_*A*_ ≈ *q*_*C*_, i.e. *q*_*A*_/*q*_*C*_ ≈ 1, the event horizon is less than 500 m away from CV1, requiring maybe one message hop for transmitting the alert to CV2. Finally, in Fig 9(a) and 9(b), we observe that *q*_*F*_ has no impact on the location of the event horizon (as expected). Thus, for the specific parameters considered in this scenario, the discussion of *minimum* message hopping is moot as far as the event horizon is concerned.

#### 6.5.2 Minimum penetration rate requirements

Similar insights can be discussed from the perspective of penetration rate of CVs and V2I communication, with the *strong* caveat that the developed analytical solutions pertain to a scenario with two CVs. We define the minimum CV penetration rate (*p*_*min*_) required to mitigate delay as the ratio of density of Connected Vehicles to vehicular density of traffic state *A*. Mathematically,

$$\begin{array}{c} p_{min}=\frac{1/\xi_{N}^{\prime }}{k_{A}} \end{array}$$ (12)

where, $\xi_{N}^{\prime }$ represents the maximum spacing between CV1 and CV2, so $1/\xi_{N}^{\prime }$ represents the minimum density of CVs that mitigates delay. We reason that to determine the minimum penetration rate, only one additional Connected Vehicle (e.g., CV2) needs to be located downstream of the null horizon to have a potential positive impact on the traffic macrostate *D*^∞^ (assuming V2I communication). Of course, such an analysis requires the strong assumption that CVs are distributed perfectly uniformly in traffic flow, which must be relaxed in future works. However, even with this strong assumption, we are able to obtain some illuminating insights. We now discuss these with the knowledge that they apply to the specific parameters representative of typical traffic flows that we have used in the analysis.

**Fig 10:**
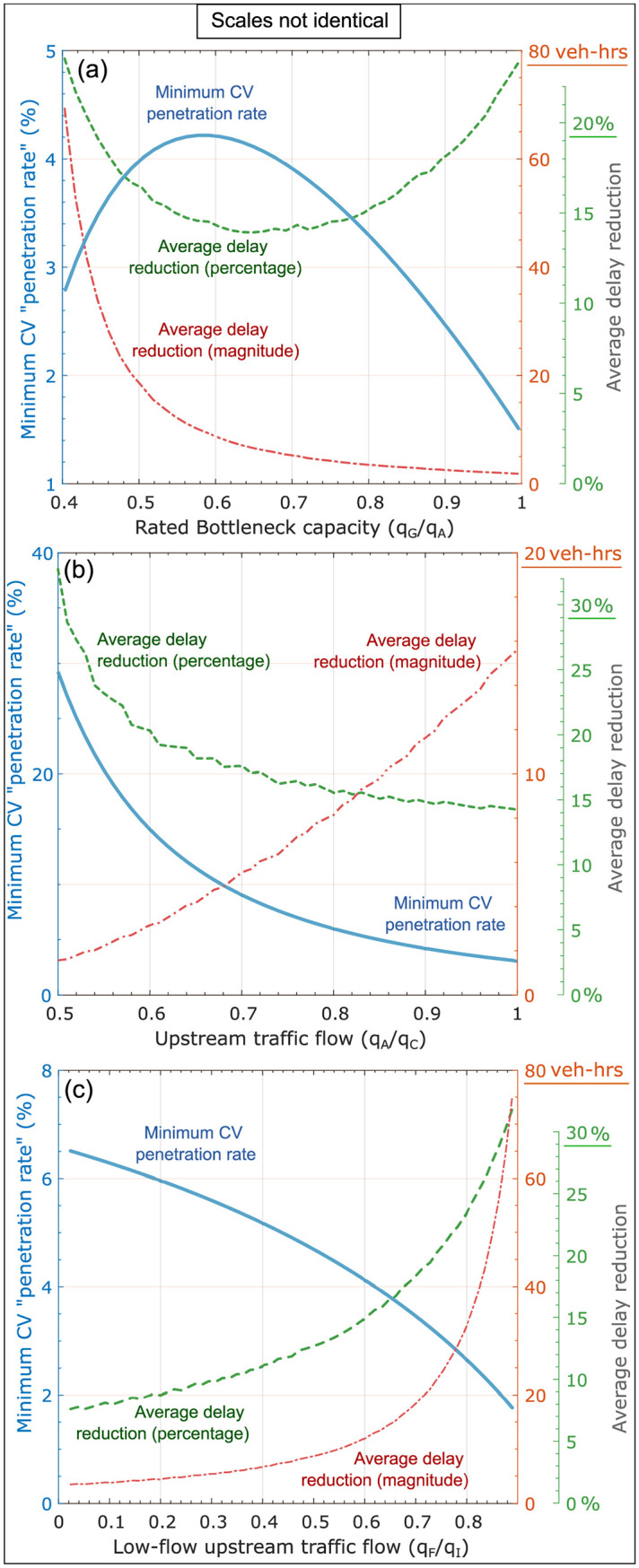
Minimum penetration rate requirements corresponding to the presence of at least one connected vehicle downstream of the null horizon. The presence of this Connected Vehicle (CV2) has the potential to reduce delay using the presented control actions. Average magnitude and percentage reductions corresponding to minimum penetration rate are shown.

Fig 10 shows the various minimum penetration rates as well as the associated average delay reduction for the three different scenarios discussed in Sections 6.2–6.4. The average delay reduction is calculated by averaging ΔD over the entire zone of influence. By examining Fig 10(a) we note that when the ratio *q*_*G*_/*q*_*A*_ is relatively small, then we can obtain *significant delay reductions* (≈ 70 veh-hrs or 25%) *even with very low penetration rates* (≈ 3%). This is evident from Fig 7 where low *q*_*G*_/*q*_*A*_ ratios correspond to significant delay reductions and large zones of influence. On the other hand, when the rated bottleneck capacity *q*_*G*_ is relatively large (i.e. *q*_*G*_/*q*_*A*_ → 1), then penetration rate requirements are also low, but the magnitude of average delay reduction is insignificant (≈ 2 veh-hrs). These quantitative results confirm our qualitative understanding that limited delay reduction should be expected since the bottleneck can only just about accommodate the flow from the upstream traffic state A.

Next, in Fig 10(b), we examine the minimum penetration rate required to mitigate delay for various values of upstream traffic flow *q*_*A*_. When the ratio *q*_*A*_/*q*_*C*_ → 1, we observe that the null horizon is farther upstream. This indicates that a low penetration rate (≈ 4%) is required to produce delay reductions (≈ 15 veh-hrs or 15%). In fact, for such large flows it is difficult to mitigate delays as the freeway is operating at near maximum capacity. This a direct result of the short extent of the zone of influence.

Finally in Fig 10(c), we see that as *q*_*F*_/*q*_*I*_ → 1, the effects of this larger flow rate of the low-flow upstream traffic state *F* provides the opportunity for CV2’s actions to significantly reduce average delays. Specifically, as *q*_*F*_ approaches *q*_*I*_, even low penetration rates (≈ 2%) can produce significant delay reductions in magnitude (≈ 80 veh-hrs) *and* percentage (≈ 35%). On the other hand, higher minimum penetration rates at low values of *q*_*F*_ have limited impact, since the low flow rate implies that few vehicles are impacted and the delay reductions are relatively insignificant.

A **key insight** obtained from these observations is that, in many traffic flow scenarios of interest, even low penetration rates can significantly reduce total vehicular delay, within the constraints of the parameters used in the analysis. This and similar insights can help make a decision as to which penetration rates are beneficial in specific scenarios to mitigate delays. In the case of limited bandwidth for V2I communications, we can use this quantitative analysis to determine which regions and traffic states would benefit from receiving direct information from CVs, such that we obtain the most delay reductions for most vehicles. Of course, higher penetration rates could leverage the zones of influence to develop more advanced delay mitigation strategies. The expansion of the discussed ideas to multiple connected vehicles is currently being investigated by the authors.

## 7 Concluding remarks and future work

The presented work seeks to answer an important question concerning the distribution of traffic macrostate information using connected vehicles: *where and to whom should this information be delivered?* Our work introduces the notion of zones of influence and event horizons on roadways, with an exemplary study demonstrating these concepts in the context of traffic jam or queue formation resulting from capacity drop at fixed freeway bottlenecks. As discussed above, we show that a pair of connected vehicles that possess specific event-triggered control policies can positively impact the traffic macrostate in only a specific region, i.e., the Zone of Influence. The range of the ZOI is a function on the spatial distance between the two connected vehicles, the existing traffic macrostate, and the control policies available for the connected vehicles to choose from. While the discussion was carried out in the context of capacity drop and queue formation at fixed freeway bottlenecks, the introduced concepts are equally applicable across a wide range of traffic flow scenarios.

We have also demonstrated and analytically calculated the existence of event and null horizons, i.e. spatial locations beyond which a connected vehicle has no positive impact with a known control policy. Our work shows that communicating macroscopic-scale information about the traffic state, such as the presence of congestion caused by capacity drop at a freeway bottleneck, has limited or no utility outside the zone of influence for a known event-triggered control policy. In some spatial locations outside the ZOI, such as downstream of the event horizon (i.e. closer to the bottleneck), communication with connected vehicles may be beneficial but only as a means to propagate the message further upstream to connected vehicles which are located within the ZOI.

### 7.1 Summary of results

Table 3 provides a summary of the discussions, including both qualitative and quantitative results. One of the primary interests of practitioners and stakeholders is to mitigate traffic delays when and where they occur. Knowledge of the zones of influence and associated traffic states can help direct information and resources to the appropriate regions that can generate quantifiable positive impact. From the summary presented in the Table 3, we note that large average delay reductions correspond to large influential spaces that enable CV2 to significantly impact traffic flow states. Importantly, such large average delay reductions also correspond directly to low CV penetration rates, which is a positive outcome. Further, by observing the ratios of interest, we can direct information and resources to regions where these traffic conditions are dominant. For example, it is useful to dedicate information transmission resources towards regions where the ratio *q*_*G*_/*q*_*A*_ is typically small, or where the ratios *q*_*A*_/*q*_*C*_ or *q*_*F*_/*q*_*I*_ are typically large. These situations correspond to scenarios where rated bottleneck capacity is less than upstream traffic flow, or where upstream traffic flow is operating at maximal capacity, or where the low-flow states are close to the reduced bottleneck capacities. While these qualitative results may appear intuitive and obvious in retrospect, the primary contribution of this work is to demonstrate that we can also obtain analytical expressions and a quantitative understanding of these scenarios.

However, the spatial locations of the event and null horizons, and by consequence the spatial extent of the ZOI, can change in several scenarios. Specifically, these characteristics are expected to vary if: (a) additional connected vehicles are present in the traffic stream, (b) a different macroscopic quantity or traffic macrostate is of interest, and subsequent analysis is conducted to examine if CVs can positively impact this new traffic macrostate, and (c) a wider range of event-triggered control policies are available for the connected vehicles to choose from. The authors expect each of these rich variations of the presented problem to provide additional insightful details of traffic flow evolution that will benefit traffic system managers and designers of connected vehicle control policies. Each of these variations provide an opportunity for extending the notion of zones of influence to increasingly useful traffic scenarios. Our future work will not only examine alternative event-triggered policies for CV2, but also different trigger protocols for CV1 as well. This may also enable an evaluation of the scalability of the proposed approach, using both analytical and numerical approaches, to platoons or scenarios with multiple connected vehicles that are spatially distributed across the roadway. The presented work is a building block that potentially enables the creation of a scalable, analytical formulation of the zone of influence with applications to diverse traffic scenarios in the long-term.

## Supporting information

S1 Appendix Analytical expression for total vehicular delay in the absence of connected vehicles (*D*_0_). (PDF)

S2 Appendix Analytical expression for total vehicular delay in the presence of connected vehicles CV1 and CV2 (*D*_1_). (PDF)
