# Supplementary material for: Mitigating delay due to capacity drop near freeway bottlenecks: Zones of influence of connected vehicles
Source: PLoS One. 2024 Jun 5;19(6):e0301188. doi: 10.1371/journal.pone.0301188 (PMC11152315; doi:10.1371/journal.pone.0301188)
Supplement: S1 Appendix — (PDF) [file pone.0301188.s001.pdf]

Appendix A: Analytical Expression for Total Vehicular Delay in the Absence of Connected Vehicles ($\mathcal{D}_0$)

Here, we obtain analytical expressions for the total vehicular delay at a fixed freeway bottleneck in the absence of any corrective action taken by connected vehicles. The total vehicular delay in absence of connected vehicles ($\mathcal{D}_0$) is evaluated by calculating the shaded region in Fig. 11(b) between the virtual arrival and departure curves, i.e. summing the area of regions R_1 and R_2, and using traffic parameters such as flow rates in different states and location of transitions between traffic states. Additional details about the traffic states can be found in the fundamental diagram of traffic flow shown in Fig. 2.

Fig 11. Analytical calculation of total vehicular delay for capacity drop scenario at fixed freeway bottleneck in absence of connected vehicles. (a) Time-space diagram indicates various traffic states, interface points (α, β, γ, and δ), and parameters used in delay calculations. (b) $N - t$ curve with shaded region indicating total vehicular delay ($\mathcal{D}_0$), which is evaluated by summing the areas of shaded regions R_1 and R_2.

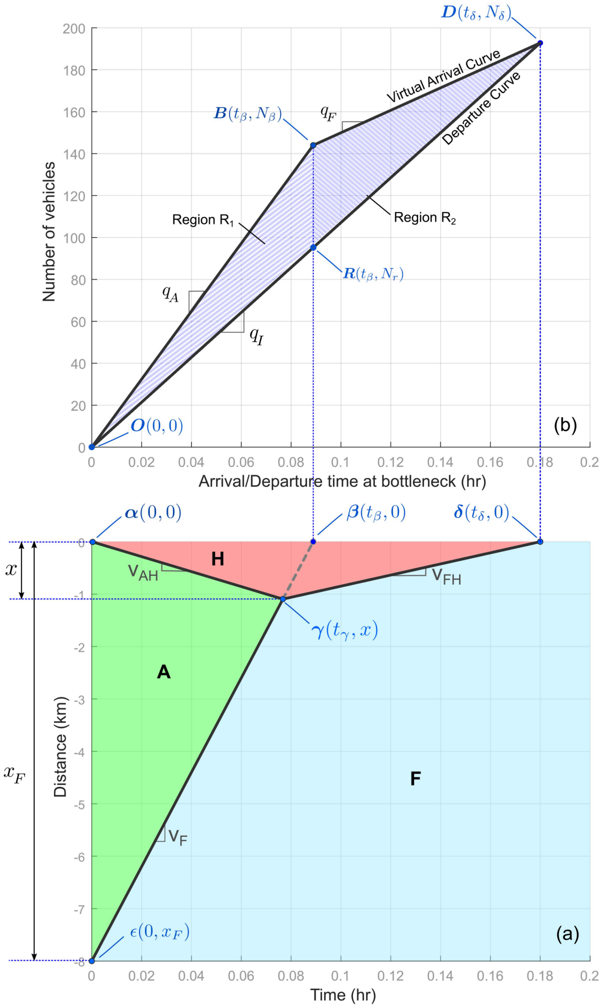

We begin by focusing on the time-space diagram in Fig. 11(a) to determine the various unknown values such as t_β, t_γ, x, and t_δ, in terms of the known traffic flow states and velocities. It is also assumed that the location x_F of the low-flow free-flow state F at time $t = 0$ is known. Before we determine the actual vehicular delay, it is helpful to determine some intermediate unknown quantities in terms of known traffic flow parameters. For example, to evaluate t_γ, we focus on Point γ in Fig. 11(a) and realize that:

$$v_{AH} \cdot t_\gamma + v_F \cdot t_\gamma = x_F$$
$$\text{or, } t_\gamma = \frac{x_F}{v_{AH} + v_F} \tag{A.1}$$

Additionally, substituting t_γ to obtain the expression for x:

$$x = v_{AH} \cdot t_\gamma = \left(\frac{v_{AH}}{v_{AH} + v_F} \right) x_F \tag{A.2}$$

Shifting our attention to Point β in Fig. 11(a), we notice that:

$$v_F \cdot t_\beta = x_F \implies t_\beta = \frac{x_F}{v_F} \tag{A.3}$$

Moving on to Point δ, we observe:

$$v_{AH} \cdot t_\gamma - v_{FH} \cdot (t_\delta - t_\gamma) = 0$$
$$\text{or, } t_\delta = \left(\frac{v_{AH} + v_{FH}}{v_{FH}} \right) \cdot t_\gamma$$
$$\text{or, substituting for } t_\gamma,\ t_\delta = \left(\frac{v_{AH} + v_{FH}}{v_{FH}} \right) \cdot \left(\frac{x_F}{v_{AH} + v_F} \right) \tag{A.4}$$

With this information, we can begin to calculate the total vehicular delay $\mathcal{D}_0$ at the fixed freeway bottleneck in the absence of connected vehicles using the areas of Regions R_1 and R_2 in Fig. 11(b):

$$Ar(R_1) = Area(\Delta ORB) = \frac{1}{2} (N_\beta - N_r) \cdot t_\beta$$
$$= \frac{1}{2} (q_A t_\beta - q_I t_\beta) \cdot t_\beta$$
$$\text{so, } Ar(R_1) = \frac{1}{2} (q_A - q_I) \cdot t_\beta^2 \tag{A.5}$$

Similarly, we can evaluate the area of Region R_2 with reference to Fig. 11(b) as follows:

$$Ar(R_2) = Area(\Delta DRB) = \frac{1}{2} (N_\beta - N_r) \cdot (t_\delta - t_\beta)$$
$$= \frac{1}{2} (q_A t_\beta - q_I t_\beta) \cdot (t_\delta - t_\beta) \tag{A.6}$$

Then, the total vehicular delay is obtained by adding the right hand sides of Equations (A.5) and (A.6) to get:

$$\begin{aligned}\mathcal{D}_0 &= \frac{1}{2}\left(q_A - q_I\right)\cdot t_\beta^2 + \frac{1}{2}\left(q_A t_\beta - q_I t_\beta\right)\cdot\left(t_\delta - t_\beta\right)\\ &= \frac{1}{2}\left(q_A - q_I\right)\cdot t_\beta \cdot t_\delta\end{aligned}$$

or, substituting for t_β and t_δ from Equations (A.3) and (A.4),

$$\mathcal{D}_0 = \frac{1}{2}\left(q_A - q_I\right)\cdot\left\{\frac{v_{AH} + v_{FH}}{v_F v_{FH}\left(v_{AH} + v_F\right)}\right\}\cdot x_F^2 \tag{7}$$

Thus, the total vehicular delay in the absence of any corrective actions by connected vehicles is given by:

$$\mathcal{D}_0(\theta) = \frac{1}{2}\left(q_A - q_I\right)\cdot\left\{\frac{v_{AH} + v_{FH}}{v_F \cdot v_{FH}\cdot\left(v_{AH} + v_F\right)}\right\}\cdot x_F^2 = \lambda_0 \cdot x_F^2 \tag{8}$$

where $\theta = \{q_A, q_I, x_F, v_F, v_{AH}, v_{FH}\}$ represents the set of all parameters on the right hand side, which are defined in Figs. 2 and 11, and $\lambda_0 = \frac{1}{2}\left(q_A - q_I\right)\cdot\left\{\frac{v_{AH} + v_{FH}}{v_F \cdot v_{FH}\cdot\left(v_{AH} + v_F\right)}\right\}$.
