# Supplementary material for: Mitigating delay due to capacity drop near freeway bottlenecks: Zones of influence of connected vehicles
Source: PLoS One. 2024 Jun 5;19(6):e0301188. doi: 10.1371/journal.pone.0301188 (PMC11152315; doi:10.1371/journal.pone.0301188)
Supplement: S2 Appendix — (PDF) [file pone.0301188.s002.pdf]

# Appendix B: Analytical Expression for Total Vehicular Delay in the Presence of Connected Vehicles CV1 and CV2 ($\mathcal{D}_1$)

In this section, we obtain analytical expressions for the total vehicular delay ($\mathcal{D}_1$) at the fixed freeway bottleneck when the pair of connected vehicles CV1 and CV2 use an event-triggered action policy to mitigate the effects of capacity drop at the bottleneck. The total vehicular delay is calculated by adding the areas of the shaded regions $R_1$, $R_2$, $R_3$, and $R_4$, as indicated in Fig. 12(b):

$$\mathcal{D}_1(x_1, x_2, \theta') = \mathrm{Ar}(R_1) + \mathrm{Ar}(R_2) + \mathrm{Ar}(R_3) + \mathrm{Ar}(R_4) \tag{B.1}$$

where $x_1$ and $x_2$ denote the positions of connected vehicles CV1 and CV2 at time $t = 0$ hr., respectively, and $\theta'$ represents the set of all traffic flow parameters needed to evaluate the delay.

**Fig 12. Analytical calculation of total vehicular delay for capacity drop scenario**. This analysis is performed at fixed freeway bottleneck when connected vehicles use event-triggered control policy to take corrective action to mitigate the queue formation. (a) Time-space diagram indicates various traffic states, interface points, and parameters used in delay calculations. (b) $N-t$ curve with shaded region indicating total vehicular delay ($\mathcal{D}_1$), which is evaluated by summing the areas of shaded regions $R_1$, $R_2$, $R_3$ and $R_4$.

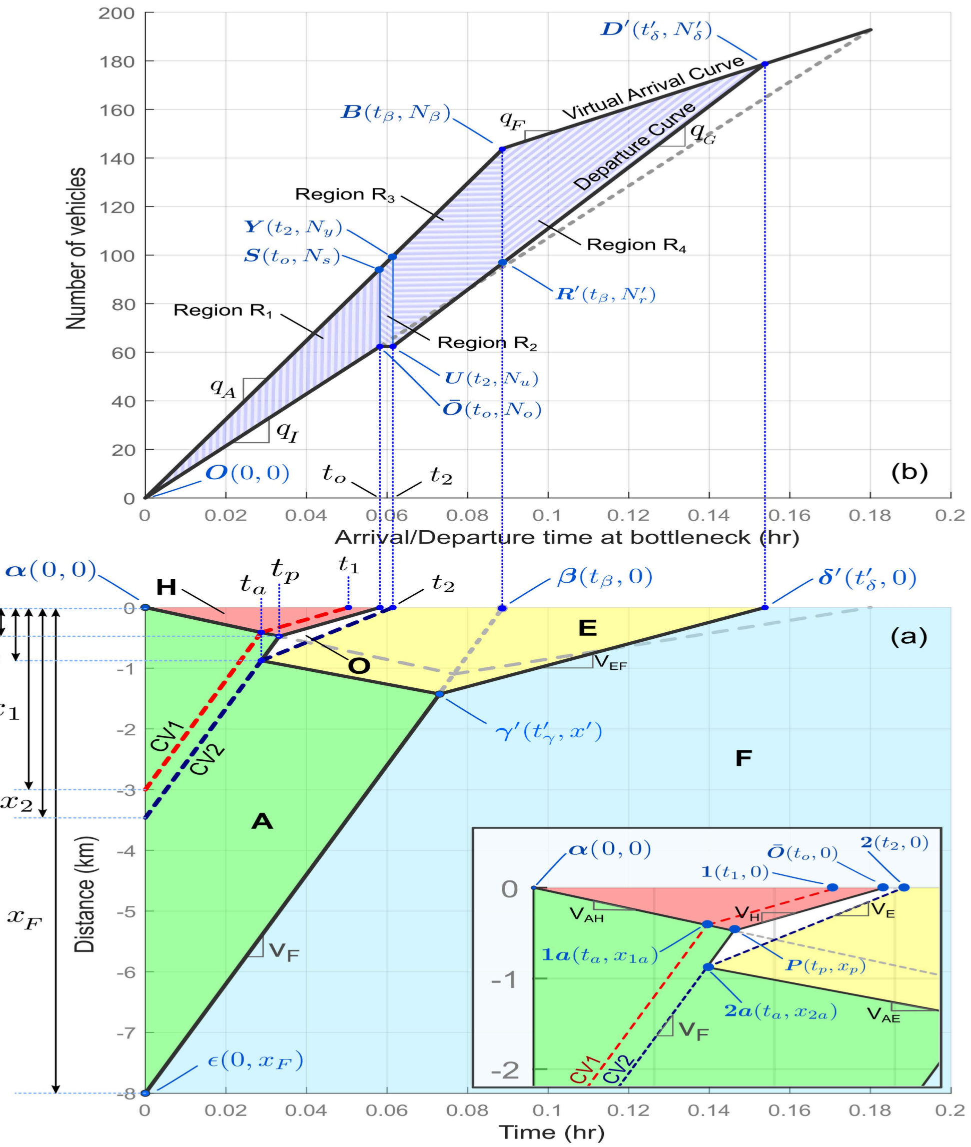

Before the area of the shaded regions can be evaluated, we must determine expressions for some unknown quantities in terms of the known traffic flow parameters. Specifically, we will focus on identifying the coordinates of the various points included in Fig. 12, and for the sake of brevity, will only evaluate those quantities that are required to evaluate the macroscopic vehicular delay. Beginning with Point $\boldsymbol{1a}$ (see inset in Fig. 12(a)), we express the time $t_a$, which represents the time instant when CV1 enters the queue at the bottleneck and sends an alert signal to upstream vehicles, in terms of other known quantities:

$$\begin{aligned} v_{AH} \cdot t_a + v_F \cdot t_a &= x_1 \\ \implies \quad t_a &= \frac{x_1}{v_{AH} + v_F} \end{aligned} \tag{B.2}$$

where $v_{AH}$ and $v_F$ are defined in reference to Fig. 2. We note that $t_a$ also denotes the time instant at which CV2 receives the alert signal from CV1, as it is assumed that the signal transmission is instantaneous in nature. Additionally, using Point $\boldsymbol{2a}$ we can evaluate the position $x'$ of CV2 when it receives the alert signal from CV1 as follows:

$$\begin{aligned} x_2 - x_2' &= v_F \cdot t_a \\ \implies x_2' &= x_2 - v_F \cdot t_a \end{aligned} \tag{B.3}$$

Moving to Point $\boldsymbol{P}$, we identify the time $t_p$, which represents the time at which the vehicle preceding CV2 joins the queue at the bottleneck:

$$\begin{aligned} v_{AH} \cdot t_p + v_F \cdot t_p &= x_2 \\ \implies \quad t_p &= \frac{x_2}{v_{AH} + v_F} \end{aligned} \tag{B.4}$$

Next, we observe Point $\bar{\boldsymbol{O}}(t_o, 0)$. As a result of CV2 initiating corrective action based on CV1's alert signal received at $t_a$, an open traffic flow state $O(q_O = 0, k_O = 0)$ forms immediately downstream of CV2. The time $t_o$ corresponds to the when this open traffic state $O$ first reaches the bottleneck. Using Points $\boldsymbol{\alpha}$, $\boldsymbol{P}$ and $\bar{\boldsymbol{O}}$, we get:

$$\begin{aligned} v_{AH} \cdot t_p - v_H \cdot (t_o - t_p) &= 0 \\ \text{or,} \quad t_o &= \left( \frac{v_{AH} + v_H}{v_H} \right) \cdot t_p \end{aligned}$$

or, substituting for $t_p$ from Equation (B.4),

$$t_o = \frac{1}{v_H} \cdot \left( \frac{v_{AH} + v_H}{v_{AH} + V_F} \right) \cdot x_2 \tag{B.5}$$

Now, we shift focus to Point $\boldsymbol{2}(t_2, 0)$, which corresponds to the event of CV2 reaching the fixed freeway bottleneck. To find the time $t_2$, we use Points $\boldsymbol{2a}$ and $\boldsymbol{2}$ as follows:

$$\begin{aligned} & & x_2' &= v_E \cdot (t_2 - t_a) \\ &\text{or,} & t_2 &= \frac{1}{v_E} \cdot (x_2' + v_E \cdot t_a) \\ &\text{or substituting } x_2' \text{ from Equation (B.3),} & t_2 &= \frac{1}{v_E} \cdot (x_2 - v_F \cdot t_a + v_E \cdot t_a) \\ &\text{and substituting } t_a \text{ from Equation (B.2),} & t_2 &= \frac{1}{v_E} \cdot \left\{ x_2 - \left( \frac{v_F - v_E}{v_{AH} + v_F} \right) \cdot x_1 \right\} \end{aligned} \tag{B.6}$$

Moving onward to Point $\boldsymbol{\gamma'}(t'_\gamma, x')$, in combination with Point $\mathbf{2a}$, we determine the time $t'_\gamma$) when the first vehicle transitions from traffic state $F$ to state $E$, as follows:

$$(v_F + v_{AE}) \cdot (t'_\gamma - t_a) = x_F - v_F \cdot t_a - x'_2$$

or, substituting from (B.3),

$$(v_F + v_{AE}) \cdot (t'_\gamma - t_a) = x_F - v_F \cdot t_a - (x_2 - v_F \cdot t_a)$$

or,

$$t'_\gamma = t_a + \left( \frac{x_F - x_2}{v_F + v_{AE}} \right)$$

and substituting from (B.2),

$$t'_\gamma = \left( \frac{x_1}{v_{AH} + v_F} \right) + \left( \frac{x_F - x_2}{v_F + v_{AE}} \right) \tag{B.7}$$

and additionally,

$$\begin{aligned} x' &= x_F - v_F \cdot t'_\gamma \\ \implies x' &= x_F - v_F \cdot \left\{ \left( \frac{x_1}{v_{AH} + v_F} \right) + \left( \frac{x_F - x_2}{v_F + v_{AE}} \right) \right\} \end{aligned} \tag{B.8}$$

Now, we determine the time coordinate for when the state $F$ reaches the fixed freeway bottleneck by observing Point $\boldsymbol{\delta'}(t'_\delta, 0)$ and $\boldsymbol{\gamma'}(t'_\gamma, x')$ as follows:

$$x' - v_{EF} \cdot (t'_\delta - t'_\gamma) = 0$$

or, substituting from Equation (B.8),

$$x_F - v_F \cdot t'_\gamma - v_{EF} \cdot (t'_\delta - t'_\gamma) = 0$$

or,

$$t'_\delta = \frac{1}{v_{EF}} \left( x_F - (v_F - v_{EF}) \cdot t'_\gamma \right) \tag{B.9}$$

With these variables known in the form of traffic flow parameters, we can leverage Fig. 12(b) to determine the total vehicular delay $\mathcal{D}_1$ in the presence of mitigating actions of connected vehicles CV1 and CV2 as indicated in Equation (B.1) and repeated here for convenience:

$$\mathcal{D}_1(x_1, x_2, \theta') = \text{Ar}(R_1) + \text{Ar}(R_2) + \text{Ar}(R_3) + \text{Ar}(R_4)$$

where $x_1$ and $x_2$ denote the positions of connected vehicles CV1 and CV2 at time $t = 0$ hr., respectively, and $\theta'$ represents the set of all traffic flow parameters needed to evaluate the delay.

We will now evaluate the areas of each of the individual shaded regions. Beginning with **Region** $R_1$, we find that:

$$\begin{aligned} \text{Ar}(R_1) = \text{Ar}(\triangle O\bar{O}S) &= \frac{1}{2} \cdot (N_s - N_o) \cdot t_o \\ &= \frac{1}{2} \cdot (q_A - q_I) \cdot t_o^2 \end{aligned}$$

or, substituting from Equation (B.5),

$$Ar(R_1) = \frac{1}{2} \cdot (q_A - q_I) \cdot \left\{ \frac{1}{v_H} \cdot \left( \frac{v_{AH} + v_H}{v_{AH} + v_F} \right) \right\}^2 \cdot x_2^2$$

$$\text{or, } \operatorname{Ar}(R_1) = \frac{1}{2} \cdot \left\{ (q_A - q_I) \cdot c_1^2 \right\} \cdot x_2^2 \tag{B.10}$$

where $c_1 = \dfrac{1}{v_H} \cdot \left( \dfrac{v_{AH} + v_H}{v_{AH} + V_F} \right)$. 1063

Next, the **area of shaded region** $R_2$ is given by the area of the trapezoid $SYU\bar{O}$ as follows:

$$\operatorname{Ar}(R_2) = \operatorname{Ar}(SYU\bar{O}) = \frac{1}{2} \cdot \Big\{ (N_s - N_o) + (N_y - N_u) \Big\} \cdot (t_2 - t_o)$$

$$\begin{aligned} \text{or, } \operatorname{Ar}(R_2) &= \frac{1}{2} \cdot \Big\{ (q_A - q_I) \cdot t_o + (q_A \cdot t_2 - q_I \cdot t_o) \Big\} \cdot (t_2 - t_o) \\ &= \frac{1}{2} \cdot \Big\{ q_A \cdot (t_2 + t_o) - 2\, q_I \cdot t_o \Big\} \cdot (t_2 - t_o) \end{aligned}$$

$$\implies \operatorname{Ar}(R_2) = \frac{1}{2} \cdot q_A \cdot (t_2^2 - t_o^2) - q_I \cdot t_2 \cdot t_o + q_I \cdot t_o^2 \tag{B.11}$$

Substituting expressions for $t_o$ and $t_2$ from Equations (B.5) and (B.6), respectively, we get:

$$\begin{aligned} \operatorname{Ar}(R_2) = {} & \frac{1}{2} \cdot q_A \cdot \left[ \left\{ \frac{1}{v_E} \cdot x_2 - \frac{1}{v_E} \cdot \left( \frac{v_F - v_E}{v_{AH} + v_F} \right) \cdot x_1 \right\}^2 - \left\{ \frac{1}{v_H} \cdot \left( \frac{v_{AH} + v_H}{v_{AH} + V_F} \right) \cdot x_2 \right\}^2 \right] \\ & - q_I \cdot \left\{ \frac{1}{v_E} \cdot x_2 - \frac{1}{v_E} \cdot \left( \frac{v_F - v_E}{v_{AH} + v_F} \right) \cdot x_1 \right\} \cdot \left\{ \frac{1}{v_H} \cdot \left( \frac{v_{AH} + v_H}{v_{AH} + V_F} \right) \cdot x_2 \right\} \\ & + q_I \cdot \left\{ \frac{1}{v_H} \cdot \left( \frac{v_{AH} + v_H}{v_{AH} + V_F} \right) \cdot x_2 \right\}^2 \end{aligned}$$

$$\begin{aligned} \implies \operatorname{Ar}(R_2) = {} & \frac{1}{2} \cdot q_A \cdot \left\{ (c_2 \cdot x_2 - c_3 \cdot x_1)^2 - c_1^2 \cdot x_2^2 \right\} \\ & - q_I \cdot \left\{ (c_2 \cdot x_2 - c_3 \cdot x_1) \cdot c_1 \cdot x_2 \right\} + q_I \cdot \left\{ c_1^2 \cdot x_2^2 \right\} \end{aligned} \tag{B.12}$$

where $c_2 = \dfrac{1}{v_E}$, $c_3 = \dfrac{1}{v_E} \cdot \left( \dfrac{v_F - v_E}{v_{AH} + v_F} \right)$, and which, after some additional simplification, yields the area of shaded region $R_2$ as a function of connected vehicle initial positions $x_1$ and $x_2$ as follows:

$$\begin{aligned} \operatorname{Ar}(R_2) = {} & \left\{ \frac{1}{2} \cdot q_A \cdot c_3^2 \right\} \cdot x_1^2 + \left\{ \frac{1}{2} \cdot q_A \cdot (c_2^2 - c_1^2) - q_I \cdot (c_3 \cdot c_1 - c_1^2) \right\} \cdot x_2^2 \\ & + \Big\{ q_I \cdot c_1 \cdot c_3 - q_A \cdot c_2 \cdot c_3 \Big\} \cdot x_1 \cdot x_2 \end{aligned} \tag{B.13}$$

Next, the **area of shaded region** $R_3$ is given by the area of the trapezoid $YUR'B$ as follows:

$$
\begin{aligned}
\mathrm{Ar}(R_3) &= \frac{1}{2} \cdot \Big\{ (N_y - N_u) + (N_\beta - N'_r) \Big\} \cdot (t_\beta - t_2) \\
&= \frac{1}{2} \cdot \Bigg\{ (q_A \cdot t_2 - q_I \cdot t_o) + (q_A \cdot t_\beta - (q_I \cdot t_o + q_G \cdot (t_\beta - t_2))) \Bigg\} \cdot (t_\beta - t_2) \\
&= \frac{1}{2} \cdot \Bigg\{ q_A \cdot (t_\beta^2 - t_2^2) - 2 \cdot q_I \cdot t_o \cdot (t_\beta - t_2) - q_G \cdot (t_\beta^2 - t_2^2) \Bigg\} \\
&= \frac{1}{2} \cdot \Bigg\{ q_A \cdot \Big( c_4^2 x_F^2 - (c_2 x_2 - c_3 x_1)^2 \Big) - 2 q_I \cdot c_1 x_2 \cdot \Big( c_4 x_F - (c_2 x_2 - c_3 x_1) \Big) \\
&\quad - q_G \cdot \Big( c_4^2 x_F^2 - (c_2 x_2 - c_3 x_1)^2 \Big) \Bigg\}
\end{aligned} \tag{B.14}
$$

where $c_4 = 1/v_F$, and the expressions for $t_\beta$, $t_o$, and $t_2$ are obtained from Equations (A.3), (B.5), and (B.6), respectively. The above expression, after some manipulation, can be written in terms of constants, parameters and the variable positions of the connected vehicles, as follows:

$$
\begin{aligned}
\mathrm{Ar}(R_3) = &\Big\{ \frac{1}{2} \cdot (q_A - q_G) \cdot c_4^2 \Big\} \cdot x_F^2 - \Big\{ \frac{1}{2} \cdot (q_A + q_G) \cdot c_3^2 \Big\} \cdot x_1^2 + \\
&\Big\{ -\frac{1}{2} \cdot (q_A + q_G) \cdot c_2^2 + q_I \cdot c_1 \cdot c_2 \Big\} \cdot x_2^2 \\
&+ \Bigg\{ (q_A + q_G) \cdot c_2 \cdot c_3 - q_I \cdot c_1 \cdot c_3 \Bigg\} \cdot x_1 \cdot x_2 + \\
&\Bigg\{ q_G \cdot c_2 \cdot c_4 - q_I \cdot c_1 \cdot c_4 \Bigg\} \cdot x_F \cdot x_2 - \Bigg\{ q_G \cdot c_3 \cdot c_4 \Bigg\} \cdot x_F \cdot x_1
\end{aligned} \tag{B.15}
$$

Finally, the **area of shaded region** $R_4$ is evaluated using triangle $BD'R'$ as follows:

$$
\begin{aligned}
\mathrm{Ar}(R_4) &= \frac{1}{2} \cdot (N_\beta - N'_r) \cdot (t'_\delta - t_\beta) \\
&= \frac{1}{2} \cdot \Bigg\{ q_A \cdot t_\beta - \Big( q_I \cdot t_o + q_G \cdot (t_\beta - t_2) \Big) \Bigg\} \cdot (t'_\delta - t_\beta) \\
&= \frac{1}{2} \Bigg\{ (q_A - q_G)\, c_4\, x_F - q_I\, c_1\, x_2 + q_G\, (c_2\, x_2 - c_3\, x_1) \Bigg\} (c_5\, x_F + c_6\, x_2 - c_7\, x_1 - c_4\, x_F)
\end{aligned} \tag{B.16}
$$

where, following from Equations (B.7) and (B.9), $t'_\delta = c_5 x_F + c_6 x_2 - c_7 x_1$, and $c_5 = \frac{1}{v_{EF}} \cdot \left( \frac{v_{AE} + v_{EF}}{v_F + v_{AE}} \right)$, $c_6 = \frac{1}{v_{EF}} \cdot \left( \frac{v_F - v_{EF}}{v_F + v_{AE}} \right)$, and $c_7 = \frac{1}{v_{EF}} \cdot \left( \frac{v_F - v_{EF}}{v_F + v_{AH}} \right)$. Thus,

$$
\begin{aligned}
\mathrm{Ar}(R_4) = &\left\{\frac{1}{2}\cdot(q_A - q_G)\cdot c_4\cdot(c_5 - c_4)\right\}\cdot x_F^2 + \left\{\frac{1}{2}\cdot(q_G\cdot c_2 - q_I\cdot c_1)\cdot c_6\right\}\cdot x_2^2 \\
&+\left\{\frac{1}{2}\cdot q_G\cdot c_3\cdot c_7\right\}\cdot x_1^2 + \left\{\frac{1}{2}\cdot\Big((q_A - q_G)\cdot c_4\cdot c_6 + (q_G\cdot c_2 - q_I\cdot c_1)\cdot(c_5 - c_4)\Big)\right\}\cdot x_F\cdot x_2 \\
&-\left\{\frac{1}{2}\cdot\Big((q_A - q_G)\cdot c_4\cdot c_7 + q_G\cdot c_3\cdot(c_5 - c_4)\Big)\right\}\cdot x_F\cdot x_1 \\
&-\left\{\frac{1}{2}\cdot\Big((q_G\cdot c_2 - q_I\cdot c_1)\cdot c_7 + q_G\cdot c_3\cdot c_6\Big)\right\}\cdot x_1\cdot x_2 \qquad \text{(B.17)}
\end{aligned}
$$

We have now evaluated analytical expressions for areas of all the relevant shaded regions in Fig. 12(b), so the total delay in the presence of mitigating actions of the connected vehicles is, after minor mathematical manipulation, given by:

$$
\begin{aligned}
\mathcal{D}_1(x_1, x_2, \theta') &= \mathrm{Ar}(R_1) + \mathrm{Ar}(R_2) + \mathrm{Ar}(R_3) + \mathrm{Ar}(R_4) \\
&= \lambda_1 x_F^2 + \lambda_2 x_2^2 + \lambda_3 x_1^2 + \lambda_4 x_1 x_2 + \lambda_5 x_F x_2 + \lambda_6 x_F x_1 \qquad \text{(B.18)}
\end{aligned}
$$

where,

$$
\begin{aligned}
\lambda_1 &= \frac{1}{2}\cdot(q_A - q_G)\cdot c_4\cdot c_5 \\
\lambda_2 &= \frac{1}{2}\cdot\Big(q_I\cdot c_1\cdot(c_1 - c_6) - q_G\cdot c_2\cdot(c_2 - c_6)\Big) \\
\lambda_3 &= \frac{1}{2}\cdot q_G\cdot c_3\cdot(c_7 - c_3) \\
\lambda_4 &= \frac{1}{2}\cdot\Big(2q_G\cdot c_2\cdot c_3 - (q_G\cdot c_2 - q_I\cdot c_1)\cdot c_7 - q_G\cdot c_3\cdot c_6\Big) \\
\lambda_5 &= \frac{1}{2}\cdot\Big((q_A - q_G)\cdot c_4\cdot c_6 + (q_G\cdot c_2 - q_I\cdot c_1)\cdot(c_4 + c_5)\Big) \\
\lambda_6 &= \frac{1}{2}\cdot\Big(-(q_A - q_G)\cdot c_4\cdot c_7 - q_G\cdot c_3\cdot(c_4 + c_5)\Big)
\end{aligned}
\qquad \text{(B.19)}
$$

and the constants are given by: $c_1 = \frac{1}{v_H}\cdot\left(\frac{v_{AH} + v_H}{v_{AH} + V_F}\right)$, $c_2 = \frac{1}{v_E}$, $c_3 = \frac{1}{v_E}\cdot\left(\frac{v_F - v_E}{v_{AH} + v_F}\right)$, $c_4 = \frac{1}{v_F}$, $c_5 = \frac{1}{v_{EF}}\cdot\left(\frac{v_{AE} + v_{EF}}{v_F + v_{AE}}\right)$, $c_6 = \frac{1}{v_{EF}}\cdot\left(\frac{v_F - v_{EF}}{v_F + v_{AE}}\right)$, and $c_7 = \frac{1}{v_{EF}}\cdot\left(\frac{v_F - v_{EF}}{v_F + v_{AH}}\right)$.
